# Multi-Attribute Decision Making with Einstein Aggregation Operators in Complex Q-Rung Orthopair Fuzzy Hypersoft Environments

**DOI:** 10.3390/e24101494

**Published:** 2022-10-19

**Authors:** Changyan Ying, Wushour Slamu, Changtian Ying

**Affiliations:** 1 School of Information Science and Engineering, Xinjiang University, Urumqi 830046, China; 2 Laboratory of Multi-Lingual Information Technology, Xinjiang University, Urumqi 830046, China; 3 Xinjiang Multi-Lingual Information Technology Research Center, Xinjiang University, Urumqi 830046, China; 4 Department of Computers, Shaoxing University, Shaoxing 312000, China

**Keywords:** complex q-rung orthopair fuzzy hypersoft set (Cq-ROFHSS), multi-attribute decision making, Einstein aggregation operator

## Abstract

The purpose of our research is to extend the formal representation of the human mind to the concept of the complex q-rung orthopair fuzzy hypersoft set (Cq-ROFHSS), a more general hybrid theory. A great deal of imprecision and ambiguity can be captured by it, which is common in human interpretations. It provides a multiparameterized mathematical tool for the order-based fuzzy modeling of contradictory two-dimensional data, which provides a more effective way of expressing time-period problems as well as two-dimensional information within a dataset. Thus, the proposed theory combines the parametric structure of complex q-rung orthopair fuzzy sets and hypersoft sets. Through the use of the parameter *q*, the framework captures information beyond the limited space of complex intuitionistic fuzzy hypersoft sets and complex Pythagorean fuzzy hypersoft sets. By establishing basic set-theoretic operations, we demonstrate some of the fundamental properties of the model. To expand the mathematical toolbox in this field, Einstein and other basic operations will be introduced to complex q-rung orthopair fuzzy hypersoft values. The relationship between it and existing methods demonstrates its exceptional flexibility. The Einstein aggregation operator, score function, and accuracy function are used to develop two multi-attribute decision-making algorithms, which prioritize based on the score function and accuracy function to ideal schemes under Cq-ROFHSS, which captures subtle differences in periodically inconsistent data sets. The feasibility of the approach will be demonstrated through a case study of selected distributed control systems. The rationality of these strategies has been confirmed by comparison with mainstream technologies. Additionally, we demonstrate that these results are compatible with explicit histograms and Spearman correlation analyses. The strengths of each approach are analyzed in a comparative manner. The proposed model is then examined and compared with other theories, demonstrating its strength, validity, and flexibility.

## 1. Introduction

In decision science, multi-attribute decision making (MADM) seeks to determine the best choice under different sets of attributes. There is a growing emphasis on multi-attribute decision-making in decision science, systems engineering, and management science. In addition to its applications in economics, management, engineering, and the military, its theories and methods are widely employed for investment decisions, factory site selection, college evaluation, project bidding, ranking of industrial sector development, and comprehensive economic benefits evaluation. Experts or teams of experts evaluate each alternative according to a variety of attributes, and the results can be expressed in clear numbers or in linguistic terms. In today’s challenging environment, uncertainty plays an important role in almost every decision-making process. Analyses must take uncertainty into account in order to be accurate. According to classical set theory (CST), there are only two possible outcomes for an item: either it belongs to a set, or it does not. An item’s characteristic function can be either 0 or 1. Several factors contribute to the failure of CST, including age, intelligence, and height.

As a solution to this problem, Zadeh [1] proposed the concept of Fuzzy Sets (FS). In order to solve complex problems involving uncertainty and ambiguity, the degree of membership (Mem) is assigned to the closed interval [0, 1] rather than {0, 1}. As a result, Atanasove’s intuitionistic fuzzy set (IFS) has been demonstrated to be a useful tool for describing uncertainty in MADM [2]. There are two parameters, Mem and NMem, the sum of which is less than or equal to 1, and it is more general than fuzzy sets (FS). Researchers have developed and used some theories to explain this phenomenon, including interval-valued IFSs [3] and linguistic interval-valued IFSs [4]. The above theory indicates that the sum of an individual’s Mem and NMem cannot exceed one. Research has been conducted on MADM problems in the context of FSs and IFSs, which can only be used to address decision makers’ uncertainty and ambiguity. It is important to note, however, that when complex datasets are used, uncertainty and ambiguity often accompany periodic changes in the dataset.

With the advancement of technology over the years, it has become apparent that these and similar methods are inherently limited, and cannot handle information that changes over time, such as medical diagnosis, biometrics, etc. In light of the above surveys, it can be concluded that all of the above studies are based on pairs of real numbers. Ramot et al. [5] proposed the concept of complex fuzzy sets (CFS), which extend the range of Mem beyond the set of real numbers to the unit disk of the complex plane. For solving MADM problems involving uncertain and complex information, Alkouri and Salleh [6] proposed Complex Intuitive Fuzzy Sets (CIFS). Complex Mem and complex NMem degrees characterize the CIFSs, where the sum of the real and imaginary parts is less than or equal to one. The prevalence of CIFSs is greater than that of CFSs. There are times when CIFSs can solve problems that CFSs cannot. It extends the range of Mem and NMem levels from real to complex numbers by utilizing a unit disk. In contrast to IFSs, CIFSs have the capability of handling two-dimensional information, which prevents information loss. Kumar and Bajaj [7] used distance metrics and entropy to develop complex intuitionistic fuzzy soft sets. A series of correlation coefficients and aggregation operators based on CIFS has been proposed by Garg and Rani [8]. CIFSs have been used by Rani and Garg [9] to develop a distance metric and a power aggregation operator.

Originally developed by Yager [10], a Pythagorean fuzzy set (PFS) extends the intuitionistic fuzzy set theory. As PFSs are characterized by Mem and NMem, it provides a more comprehensive and detailed description of the intuitionistic fuzzy features of the data. PFSs assign each element a degree of Mem and a degree of NMem, whose sum of squares cannot exceed 1. In realistic MADM problems, simulating uncertainty with PFSs is clearly superior to simulating uncertainty with IFSs. Ullah et al. [11] have developed a new framework for describing uncertain or unreliable information, the Complex PFS (CPFS). The CPFS has a complex-valued Mem and a complex-valued NMem, thereby allowing it to describe the complex fuzzy characteristics of the data in a more comprehensive and meticulous manner. In CPFSs, each element is assigned a complex-valued Mem degree and a complex-valued NMem degree whose sum of squares is one. According to CPFSs, the sum of the squares of the real (and imaginary) parts of the complex non-membership degree cannot exceed [0, 1].

In some real decision-making processes, the sum of the squares of the Mem and NMem of alternatives that satisfy the criteria provided by the decision maker may be greater than 1, but their sum of q-power may be equal to or less than one. As such, Yager [12] proposed a q-rung orthopair fuzzy set (q-ROFS), which is characterized by Mem and NMem degrees meeting the condition that the q-power of Mem and NMem degrees cannot exceed 1. In practical decision-making problems involving uncertain and unpredictable information, the q-ROFS is more effective and general than existing methods. In the case of q = 1 and q = 2, the q-ROFS reduces to an IFS and a PFS, respectively. Thus, IFSs and PFSs can be considered special cases of the q-ROFS. A fuzzy Bonferroni average operator based on q-rung orthopairs has been developed by Liu [13] and applied to MAGDM. Wei et al. [14] have proposed a fuzzy heronian mean operator based on q-rung orthopairs. According to Liu and Wang [15,16], several fuzzy aggregation operators have been proposed for q-rung orthopairs. The power Maclaurin symmetric mean is also developed to aggregate the interrelationships between q-ROFNs. In [17], a q-rung orthopair linguistic heronian mean operator (HM) was proposed and applied to MADM. The concept of IVq-ROFS and the IVq-ROF multiple average operators was introduced by Joshi et al. [18]. Liu et al. [19] have developed a theory of complex q-rung orthopair fuzzy sets (Cq-ROFS). The Cq-ROFS proposes that the sum of the q-th power of the real (and imaginary) part of the complex Mem degree and the q-th power of the complex NMem degree does not exceed the unit interval. Cq-ROFS is a more general format compared to CIFSs and CPFSs. A Cq-ROFS provides a useful tool for capturing the ambiguity and periodicity in human evaluation semantics in real-world decision theory. Based on aggregation operators, AHP, and TOPSIS, Garg et al. [20] proposed a complex interval q-rung fuzzy set (CIVq-ROFS). Accordingly, Ali [21] proposed complex interval-valued q-rung orthopair fuzzy hamy mean operators and their application to decision-making strategies. In contrast to Cq-ROFS, CIVq-ROFS has a broader generalization than Cq-ROFS. All of these methods are widely used in many fields. However, a major common limitation of these theories is that they are not suitable for parametric descriptions. To overcome this complexity, Molodtsov [22] proposed the pioneering concept of Soft Sets (SS), a general mathematical parameterization tool for dealing with indeterminate, ambiguous, and indeterminate components, where certain specific parameters are evaluated. The ideas of the soft set theory are further combined with other fuzzy mathematical structures to develop new models, such as Fuzzy Soft Sets (FSSs) [23], Intuitionistic Fuzzy Soft Sets [24], Pythagorean Fuzzy Soft Sets [25,26], and q-Rung orthopair fuzzy soft sets (q-ROFSSs) [27]. Taken together, they provide a rich and diverse environment for the fuzzy modeling of parametric non-crisp data.

The existing studies, however, do not provide sufficient information regarding Mem and NMem values. However, these theories are not able to handle inconsistencies and imprecise data in general. There is a tendency for popular theories to fail to address this type of problem when an attribute is composed of a number of subattributes. By replacing the single-parameter function f with a multiparameter (sub-attribute) function, Smarandache [28] developed the concept of SSs to hypersoft sets (HSS). Samarandache argues that the established HSS is capable of handling indeterminate objects in comparison with the established SS. A number of unexpected results have been achieved in recent years as a result of the HSS theory and its extensions. Zulqarnain et al. [29] therefore proposed the intuitionistic fuzzy hypersoft set IFHSS, a generalized version of IFSS. Using the developed correlation coefficient, they developed the TOPSIS method for solving the MADM problem. The authors then proposed a robust aggregator operator for intuitionistic fuzzy Hypersoft sets [30] and applied it to the selection of suppliers. Zulqarnain et al. [31] perform the basic operations as well as their appropriate details under the Pythagorean Fuzzy Hypersoft Set (PFHS). In the context of PFHS sets, they introduce the concepts of demand and possibility operators in their definition of logical operators. Reference [32] describes the use of soft class and its analogous hypersoft mapping in the diagnosis of various diseases, including brain tumors, hepatitis, and HIV, and suggests appropriate treatments and future warnings. According to Ihsan et al. [33], the Hypersoft expert set has been applied to the enterprise decision-making recruitment process. In the presence of unpredictable factors, Wang et al. [34] proposed a fuzzy interactive Einstein dynamic membership function to measure the quality of expressive service. In order to recommend drugs for allergic diseases, Saeed et al. [35] proposed a Pythagorean fuzzy hypersoft map structure. Zulqarnain [36] proposed basic operations based on Pythagorean fuzzy hypersoft sets and correlation coefficients. Khan [37] proposed the q-rung hypersoft set (q-ROFHSS) and described its basic operations. Musa and Asaad [38] defined logical operators for bipolar hypersoft sets. The concepts of complex fuzzy hypersoft sets (CFHSS) were introduced by Rahman [39], a decision system based on its decision aggregation operation was developed and applied to decision-making, and the basic theory of interval-valued fuzzy hypersoft sets was studied. Using fuzzy sets and hypersoft sets with complex plane features, the model establishes a hybrid framework. It is possible to extend the features of the fuzzy hypersoft set to the unit circle in a complex plane, making this structure more flexible and useful. 

We extend the concept of CFHSS to Cq-ROFHSS in order to address the above problems in a comprehensive manner. In addition, the set’s properties are examined. Based on the proposed Cq-ROFHSS, the sum of the q-powers of the real (imaginary) part of the Mem and the NMem degree must be less than or equal to 1. Through the ensemble, uncertainty and ambiguity are taken into account simultaneously in complex numbers. Moreover, a central component of the multi-criteria decision problem is the aggregation of satisfaction with individual criteria in order to obtain a measure of satisfaction with all criteria. This process of aggregation must be guided by the interrelationship between individual criteria and criteria organizations. The increasing complexity of practical decision problems may necessitate the modeling of these numerous types of relationships when selecting aggregation operators [40,41].

This study should investigate the following theories based on the following arguments and evidence:

(i) As a result of complex q-ROFS theory, both the uncertainty and periodicity of the source data can be effectively modeled. When the membership function values are extended to the unit circle, a wide range of membership function values is possible. Even though it is an effective tool for parametric descriptions, it has some limitations. Since its inception, the proposed theory has been superior to the Complex q-ROFS model due to its ability to address the parametric ambiguity of two-dimensional fuzzy data.
(ii) Q-ROFS contains information about Mem and NMem, but lacks information about complex Mem and complex NMem, therefore relaxing the scope of the Q-ROFS model. In various practical applications, attributes should be further subdivided into sub-attribute values in order to facilitate a better understanding.
(iii) An HSS model replaces a single-parameter function with a multiparameter function (sub-attribute); however, it cannot handle other sources of uncertainty. The periodicity of information can be adequately accounted for by a model we developed. For the parametric modeling of periodic and ambiguous data, our proposed Cq-ROFHSS theory provides a more general and constructive framework.
(iv) Although CIFHSSs and CPFHSSs have a strong capability to deal with parameter ambiguity in two-dimensional problems, their boundary scope is constrained by some strict restrictions. We are able to capture the inaccuracies arising from parameterized uncertain environments by developing a model that relaxes them. We are able to capture the inaccuracies that are present in some parametric uncertain environments while simultaneously relaxing them.
(v) The q-ROFHSS model provides an efficient mathematical structure for resolving uncertainties in parametric datasets as well as uncertainties in linear datasets. However, it is limited to a single dimension. We generalize the q-ROFHSS model by using periodic fuzzy interpretations containing inconsistent information.

Combined with the previous point, these two advantages demonstrate the concept’s generalizability.

The purpose of this paper is to propose a complex q-rung orthopair fuzzy hypersoft set (Cq-ROFHSS) in order to address these issues. Due to the combination of both the complex q-rung orthopair fuzzy set and the outstanding mathematical theory of HSS, the method provides the best of both worlds. The mathematical framework proposed allows the parametric design of periodic and fuzzy data in order to solve multi-attribute decision-making problems. Using the Cq-ROFHSS model, it can express a large number of fuzzy multi-attribute evaluations in two dimensions. Through an adjustable parameter q, it is a robust generalization of CIFHSSs and CPFHSSs. The proposed model is briefly compared with existing competing methods in order to illustrate its remarkable flexibility. In the following section, we describe the Einstein operator of the Cq-ROFHSS as well as a few other elementary algebraic operations. Using the optimal selection and application of distributed control systems, we develop two advanced decision-making algorithms and demonstrate their effectiveness. A clear histogram and Spearman correlation analysis are used to verify the reliability and functionality of the proposed strategy.

The following are a few of the most important contributions of this paper:

(i) The purpose of this article is to systematically extend the literature in order to introduce the multi-skill, most generalized mixed model Cq-ROFHSS. A two-dimensional multi-parameter inconsistent data set can be correctly modeled with this method using an ordination-based fuzzy model.
(ii) For the purpose of demonstrating the applicability issues of the proposed method, we compare the method proposed in this paper with existing methods for the example of early warning opinion system selection. Based on empirical data obtained from distributed control systems, the rationality and responsibility of the proposed technology have been confirmed.
(iii) We present a brief analysis of the MADM method, including the development of a MADM method for Cq-ROFHSSs based on the Einstein aggregation operator and the proposed score function measure. This study examines the sensitivity of the variables involved in the Einstein aggregation operator as well as their impact on the decision results. In this study, we compare and analyze the existing MADM techniques using the IFHWA, IFHWG, PFSWA, PFSWG, and Cq-ROFWA, Cq-ROFWG operators. Thus, it is demonstrated that the strategy proposed in this paper is feasible and that its results are compatible.
(iv) As demonstrated in this article, the MADM methodologies exhibit flexibility, capability, and prominence beyond contemporary decision-making methods.

In accordance with this, the remainder of the paper is organized as follows: Section 2 briefly introduces some key concepts and terminology prior to the discussion of the goal theory. In Section 3, we present the framework for the proposed Cq-ROFHSS model, as well as basic set-theoretic operations. Additionally, Einstein and other algebraic algorithms are described for the Cq-ROFHSS. Through the application of the proposed operator, Section 4 presents two decision-making algorithms for the development of Cq-ROFHSSs and illustrates their applicability through the selection of financial evaluations for construction firms. Furthermore, Section 5 discusses MADM methods, including the proposed MADM method of the Einstein aggregation operator and its feasibility. In this paper, we investigate the impact of the variables involved in the Einstein aggregation operator on the decision-making process as well as their sensitivity. The advantages and disadvantages of the Cq-ROFHSS theory over other contemporary decision-making models are then discussed by conducting a comparative study with existing MADM techniques based on IFHWA, IFHWG, PFSWA, PFSWG, Cq-ROFWA, and Cq-ROFWG. In Section 6, the article’s conclusion is summarized, and some future research areas are suggested.

**Figure 1:**
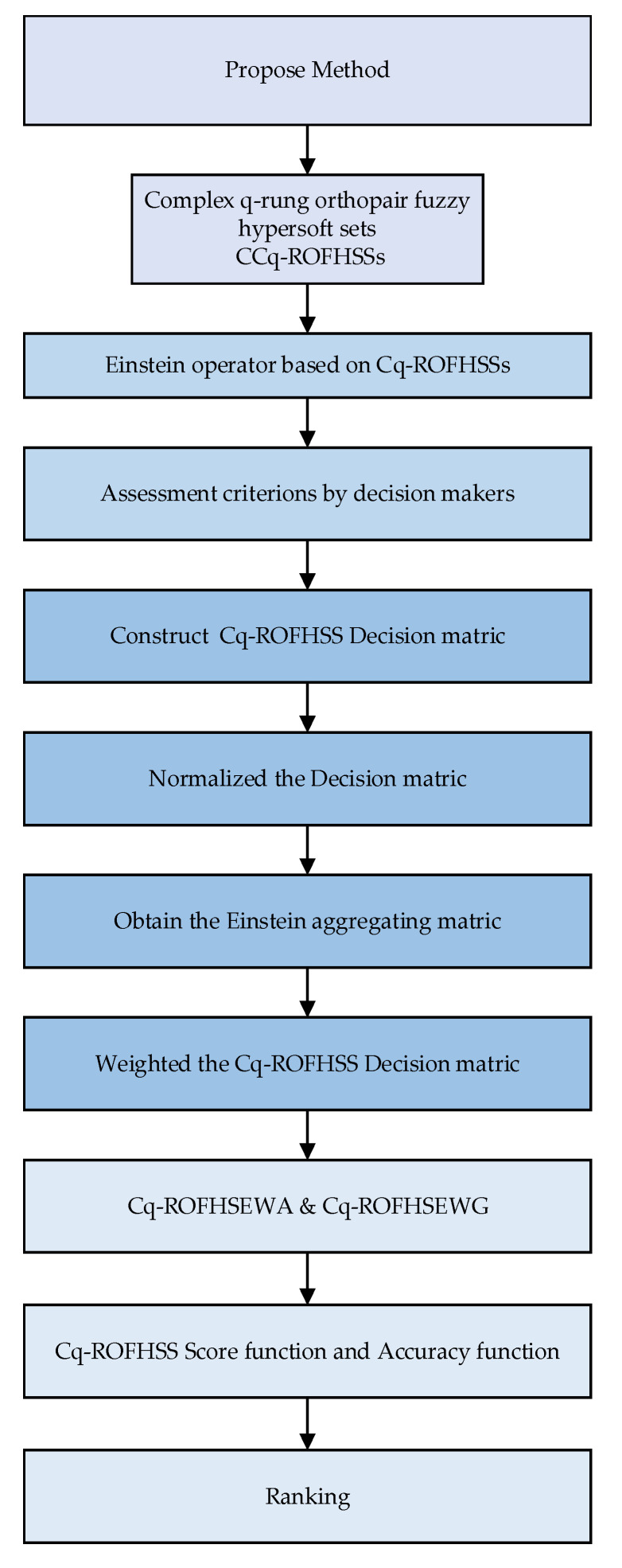
Flowchart of the proposed work in this article.

As shown in Figure 1, this research article makes a significant contribution to the field.

## 2. Preliminaries

In this section, we provide some definitions that will aid in understanding the remainder of the article. We present some basic definitions of the soft set, hypersoft set, complex q-rung orthopair fuzzy set, and complex q-rung orthopair hypersoft fuzzy set, and some operations on them that are well known in the literature.

**Definition** **1** **(see [22]).** *Let* X *be the universal set and let* Ã *be the set of parameters under consideration. Let* $P\left(X\right)$ *denote the power set of* X *and* Ã⊆Ξ *. A pair* $\left(Ђ,Ã\right)$ *is called a soft set (SS) over* X *, and its mapping is given as*

(1) $$Ђ:Ã\to P\left(X\right).$$

*A soft set may be represented by the set of ordered pairs as*

(2) $$\left(Ђ,Ã\right)=\left\{\begin{array}{l} \left.\left(ã,Ђ\left(ã\right)\right)\right|Ђ\left(ã\right)\in P\left(X\right):ã\in \Xi ,\\ Ђ\left(ã\right)=\emptyset \;if\;ã\notin Ã \end{array}\right\}$$

*In other words, the soft set is a parameterized family of subsets of the universe* X *.*

**Definition** **2** **(see [28]).** *Let* X *be the universal set and* $P\left(X\right)$ *be the power set of* X *. Consider* ${ш}_{1},{ш}_{2},\ldots ,{ш}_{n}$ *for* n≥1 *and let* n *be well-defined attributes, whose corresponding attributive values are, respectively, the set* ${Ш}_{1},{Ш}_{2},\ldots ,{Ш}_{n}$ *with* ${Ш}_{i}\cap {Ш}_{j}=\emptyset$ *, for* i≠j *and* $i,j\in \left\{1,2,\ldots ,n\right\}$ *. Assume* ${Ш}_{1}\times {Ш}_{2}\times \ldots \times {Ш}_{n}=Ě=\left\{{ě}_{1h},{ě}_{2m},\ldots ,{ě}_{nl}\right\}$ *is a collection of sub-attributes, where* 1≤h≤α, 1≤k≤β, 1≤l≤γ, *and* α,β,γ∈Ν *. Then, the pair* $\left(Ђ,Ě\right)$ *is said to be a hypersoft set over* X *, where* Ђ *is the mapping such that*

(3) $$Ђ:Ě\to P\left(X\right)$$

*where* $P\left(X\right)$ *represents the collection of all subsets of* X *. A pair* $\left(Ђ,Ě\right)$ *can be expressed as*

(4) $$\left(Ђ,Ě\right)=\left\{\left.\left(ě,Ђ(ě)\right)\right|ě\in Ě,Ђ(ě)\in P\left(X\right)\right\}$$

**Definition** **3** **(see [19]).***A complex q-rung orthopair fuzzy set (Cq-ROFS)*Q on a finite universal set X is given by:

(5) $$Q=\left\{x,\varphi_{Q}^{\prime }\left(x\right),\left.\psi_{Q}^{\prime }\left(x\right)\right|x\in X\right\},$$

*where* $\varphi_{Q}^{\prime }\left(x\right)=\varphi_{Q}\left(x\right)e^{i2\pi ({Э}_{\varphi Q\left(x\right)})}$ *and* $\psi_{Q}^{\prime }\left(x\right)=\psi_{Q}\left(x\right)e^{i2\pi ({Э}_{\psi Q\left(x\right)})}$ *represent the complex-valued membership and complex-valued non-membership degrees, with conditions:* $0\leq \varphi_{Q}^{q}\left(x\right)+\psi_{Q}^{q}\left(x\right)\leq 1$*,* $0\leq {Э}_{\varphi Q}^{q}\left(x\right)+{Э}_{\psi Q}^{q}\left(x\right)\leq 1$*, and* q≥1*. The complex q-rung orthopair fuzzy number (Cq-ROFN) is denoted by:* $Q=\left(\varphi_{Q}\left(x\right)e^{i2\pi ({Э}_{\varphi Q\left(x\right)})},\psi_{Q}\left(x\right)e^{i2\pi ({Э}_{\psi Q\left(x\right)})}\right)$.

When q=1, then Q is a complex intuition hesitant fuzzy number (CIHFN), and when q=2, then Q is a complex Pythagorean hesitant fuzzy number (CPHFN). 

**Figure 2:**
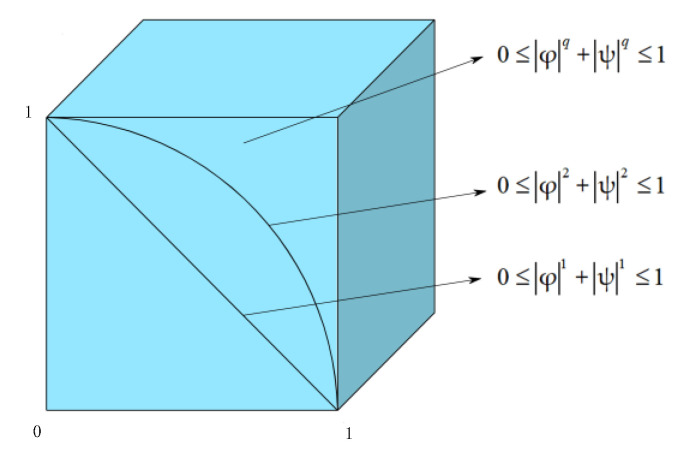
Comparisons of restrictions of the complex intuitionistic fuzzy set (CIFS), complex Pythagorean fuzzy set (CPFS), and complex q-rung orthopair fuzzy set (Cq-ROFS).

The following Figure 2 shows the comparison of the restrictions of CIFSs and CPFSs.

**Remark** **1.** *The hesitancy degree is denoted and defined by*$\theta_{Q}={\left(1-\varphi_{Q}^{q}\left(x\right)-\psi_{Q}^{q}\left(x\right)\right)}^{\frac{1}{q}}e^{i2\pi {\left(1-{Э}_{\varphi Q}^{q}(x)-{Э}_{\psi Q}^{q}(x)\right)}^{\frac{1}{q}}}$.

Similar to the operations of CIFSs and CPFSs, now we will propose the basic operations like the inclusion, complement, and equality of Cq-ROFSs.

**Remark** **2.** *Every CIF and CPFS can be considered as a Cq-ROFS but not conversely.*

**Definition** **4.** *For two Cq-ROFNs,* $Q_{1}=\left(\varphi_{1}\left(x\right)e^{i2\pi ({Э}_{\varphi_{1}\left(x\right)})},\psi_{1}\left(x\right)e^{i2\pi ({Э}_{\psi_{1}\left(x\right)})}\right)$ *and* $Q_{2}=\left(\varphi_{2}\left(x\right)e^{i2\pi ({Э}_{\varphi_{2}\left(x\right)})},\right.\left.\psi_{2}\left(x\right)e^{i2\pi ({Э}_{\psi_{2}\left(x\right)})}\right)$ *, then*

*(1)*  $Q_{1}\subseteq Q_{2}$*if*$\varphi_{1}\left(x\right)\leq \varphi_{2}\left(x\right)$*,*$\psi_{2}\left(x\right)\leq \psi_{1}\left(x\right)$*and*${Э}_{\varphi 1}\left(x\right)\leq {Э}_{\varphi 2}\left(x\right)$*,*${Э}_{\psi 2}\left(x\right)\leq {Э}_{\psi 1}\left(x\right)$.
*(2)*  $Q_{1}=Q_{2}$*if*$\varphi_{1}\left(x\right)=\varphi_{2}\left(x\right)$*,*$\psi_{2}\left(x\right)=\psi_{1}\left(x\right)$*and*${Э}_{\varphi 1}\left(x\right)={Э}_{\varphi 2}\left(x\right)$*,*${Э}_{\psi 2}\left(x\right)={Э}_{\psi 1}\left(x\right)$.
*(3)*  $Q_{1}^{c}=\left(\psi_{1}\left(x\right)e^{i2\pi ({Э}_{\psi_{1}\left(x\right)})}\right.\left.,\varphi_{1}\left(x\right)e^{i2\pi ({Э}_{\varphi_{1}\left(x\right)})}\right)$.

**Definition** **5.** *Let* X *be the universal set and* $P\left(X\right)$ *be the power set of* X *. Consider* ${ж}_{i}=\left\{{ж}_{1},{ж}_{2},\ldots ,{ж}_{n}\right\}$ *for* n≥1 *to be a set of attributes and set* ${Ж}_{i}$ *as a set of corresponding sub-attributes of* ${ж}_{i}$ *, respectively, with* ${Ж}_{i}\cap {Ж}_{j}=\emptyset$ *, for* i≠j *and* $i,j\in \left\{1,2,\ldots ,n\right\}$ *. Assume* ${Ж}_{1}\times {Ж}_{2}\times \ldots \times {Ж}_{n}=\overset{⌣}{D}=\left\{{\overset{⌣}{d}}_{1m},{\overset{⌣}{d}}_{2n},\ldots ,{\overset{⌣}{d}}_{nl}\right\}$ *is a collection of sub-attributes, where* 1≤m≤α, 1≤n≤β, 1≤l≤γ, *and* α,β,γ∈Ν *. Then, the pair* $\left(Q,\overset{⌣}{D}\right)$ *is said to be complex q-rung orthopair fuzzy hypersoft set (Cq-ROFHSS) over l, where* $Q:\overset{⌣}{D}\to P\left(X\right)$ *,* $Q\left(\overset{⌣}{D}\right)\to \left\{x\right.,{\ddot{\varphi }}_{Q\left(d\right)}\left(x\right),{\ddot{\psi }}_{Q\left(d\right)}\left(x\right),\left.x\in X\right\}$ *where* ${\ddot{\varphi }}_{Q\left(d\right)}\left(x\right)=\varphi_{Q\left(d\right)}\left(x\right)e^{i2\pi {Э}_{\varphi_{Q\left(d\right)}\left(x\right)}}$ *is the complex-valued membership, and* ${\ddot{\psi }}_{Q\left(d\right)}\left(x\right)=\psi_{Q\left(d\right)}\left(x\right)e^{i2\pi {Э}_{\psi_{Q\left(d\right)}\left(x\right)}}$ *is the complex-valued non-membership degrees such that* $Q:X\to \left[0,1\right]$ *,* $0\leq \varphi_{q\left(d\right)}^{q}\left(x\right)+\psi_{q\left(d\right)}^{q}\left(x\right)\leq 1$ *, and* $0\leq {Э}_{\varphi_{q\left(d\right)}}^{q}\left(x\right)+{Э}_{\psi_{q\left(d\right)}}^{q}\left(x\right)\leq 1$ $\left(q\geq 1\right)$ *.*

For the sake of simplicity, we write that a complex q-rung orthopair fuzzy hypersoft number (Cq-ROFHSN) can be expressed as

(6) $$A_{{}_{Q}rt}=Q_{\overset{⌣}{D}}^{x}=\left(\varphi_{Q\left(d_{t}\right)}\left(x_{r}\right)e^{i2\pi {Э}_{\varphi_{Q\left(d_{t}\right)}\left(x_{r}\right)}},\psi_{Q\left(d_{t}\right)}\left(x_{r}\right)e^{i2\pi {Э}_{\psi_{Q\left(d_{t}\right)}\left(x_{r}\right)}}\right)=\left(\varphi_{rt}e^{i2\pi {Э}_{\varphi_{rt}}},\psi_{rt}e^{i2\pi {Э}_{\psi_{rt}}}\right)$$

**Remark** **3.** *If* $0\leq \varphi_{Q\left(d\right)}^{q}\left(x\right)+\psi_{Q\left(d\right)}^{q}\left(x\right)\leq 1$ *and* $0\leq {Э}_{\varphi_{Q\left(d\right)}}^{q}\left(x\right)+{Э}_{\psi_{Q\left(d\right)}}^{q}\left(x\right)\leq 1$ $\left(q\geq 1\right)$ *are held, then all parameters of a set of attributes have no further sub-attribute. Then, Cq-ROFHSS was reduced to Cq-ROFSS.*

Ranking the alternatives scoring function of $A_{{}_{Q}rt}$ is defined in the following:

(7) $$Ş\left(A_{{}_{Q}rt}\right)=\frac{1}{4}\left(\left(1+\varphi_{rt}^{q}-\psi_{rt}^{q}\right)+\left(1+{Э}_{\varphi_{rt}}^{q}-{Э}_{\psi_{rt}}^{q}\right)\right),\;Ş\left(A_{{}_{Q}rt}\right)\in \left[0,1\right].$$

However, sometimes, the scoring function is unable to compute the two Cq-ROFHSNs. In such cases it can be difficult to decide which value is most suitable. 

An accuracy function has been introduced to overcome such difficulties:

(8) $$Ħ\left(A_{{}_{Q}rt}\right)=\frac{1}{2}\left(\left(\varphi_{rt}^{q}+\psi_{rt}^{q}\right)+\left({Э}_{\varphi_{rt}}^{q}+{Э}_{\psi_{rt}}^{q}\right)\right),\;Ħ\left(A_{{}_{Q}rt}\right)\in \left[0,1\right].$$

Thus, to compare two Cq-ROFHSNs, the subsequent ranking and comparison laws are classified as follows:

(1) If $Ş\left(A_{{}_{Q}rt}\right)>Ş\left(M_{{}_{Q}rt}\right)$, then $A_{{}_{Q}rt}>M_{{}_{Q}rt}$;
(2) If $Ş\left(A_{{}_{Q}rt}\right)=Ş\left(M_{{}_{Q}rt}\right)$, then
  if $Ħ\left(A_{{}_{Q}rt}\right)>Ħ\left(M_{{}_{Q}rt}\right)$, then $A_{{}_{Q}rt}>M_{{}_{Q}rt}$;
  if $Ħ\left(A_{{}_{Q}rt}\right)=Ħ\left(M_{{}_{Q}rt}\right)$, then $A_{{}_{Q}rt}=M_{{}_{Q}rt}$.

**Definition** **6.** *Let*$A_{{}_{Q}11}=\left(\varphi_{11}e^{i2\pi {Э}_{\varphi_{11}}},\psi_{11}e^{i2\pi {Э}_{\psi_{11}}}\right)$*, and*$A_{{}_{Q}12}=\left(\varphi_{12}e^{i2\pi {Э}_{\varphi_{12}}},\psi_{12}e^{i2\pi {Э}_{\psi_{12}}}\right)$*be two Cq-ROFHSNs over*X. *Then, some basic operations may be defined as follows:*

*(1)*  $A_{{}_{Q}11}\subseteq A_{{}_{Q}12}$*, if*$\varphi_{11}\leq \varphi_{12},{Э}_{\varphi_{11}}\leq {Э}_{\varphi_{12}},$$\psi_{11}\geq \psi_{12}$*, and*${Э}_{\psi_{11}}\geq {Э}_{\psi_{12}}$.
*(2)*  $A_{{}_{Q}11}=A_{{}_{Q}12}$*, if*$\varphi_{11}=\varphi_{12},{Э}_{\varphi_{11}}={Э}_{\varphi_{12}},$$\psi_{11}=\psi_{12}$*,*${Э}_{\psi_{11}}={Э}_{\psi_{12}}$.
*(3)*  $A_{{}_{Q}11}^{c}=\left(\psi_{11}e^{i2\pi {Э}_{\psi_{11}}},\varphi_{11}e^{i2\pi {Э}_{\varphi_{11}}}\right)$.

## 3. Weighted Aggregation Operators for Cq-ROFHSNs

### 3.1. Einstein Operations for Cq-ROFHSNs

In the fuzzy set theory, t-norm and t-conorm are used to represent fuzzy intersection and fuzzy union. Numerous t-norms and t-conorms have been proposed in the literature, including those derived from algebra, Einstein, Hamacher, Dombi, and Frank, among others. By setting certain fixed values, t-norms (ς) and t-conorms ($\varsigma^{*}$) generated by Einstein can generate algebraic and Einstein-type t-norms and t-conorms.

**Definition** **7** **(see [42]** **).** *Einstein product* ⊗ *is a t-norm and Einstein sum* ⊕ *is a t-conorm, respectively, given as*

(9) $$\varsigma \left(a,b\right)=a⊗b=\frac{ab}{1+\left(1-a\right)\left(1-b\right)};$$

(10) $$\varsigma^{*}\left(a,b\right)=a⊕b=\frac{a+b}{1+ab}.$$

Next, we examine Einstein’s operational laws for Cq-ROFHSNs, which are described in the following manner.

**Definition** **8.** *For any two Cq-ROFHSNs,*$A_{{}_{Q}11}=\left(\varphi_{11}e^{i2\pi {Э}_{\varphi_{11}}},\psi_{11}e^{i2\pi {Э}_{\psi_{11}}}\right)$*and*$A_{{}_{Q}12}=\left(\varphi_{12}e^{i2\pi {Э}_{\varphi_{12}}},\psi_{12}e^{i2\pi {Э}_{\psi_{12}}}\right)$*with*η>0.

*1*. $A_{{}_{Q}11}⊕A_{{}_{Q}12}=\left({\left(\frac{\varphi_{11}^{q}+\varphi_{12}^{q}}{1+\varphi_{11}^{q}\varphi_{12}^{q}}\right)}^{\frac{1}{q}}e^{i2\pi {\left(\frac{{Э}_{\varphi_{11}}^{q}+{Э}_{\varphi_{12}}^{q}}{1+{Э}_{\varphi_{11}}^{q}{Э}_{\varphi_{12}}^{q}}\right)}^{\frac{1}{q}}},\left(\frac{\psi_{11}\psi_{12}}{{\left(1+\left(1-\psi_{11}^{q}\right)\left(1-\psi_{12}^{q}\right)\right)}^{\frac{1}{q}}}\right)e^{i2\pi \left(\frac{{Э}_{\psi_{11}}{Э}_{\psi_{12}}}{{\left(1+\left(1-{Э}_{\psi_{11}}^{q}\right)\left(1-{Э}_{\psi_{12}}^{q}\right)\right)}^{\frac{1}{q}}}\right)}\right)$
*2*. $A_{{}_{Q}11}⊗A_{{}_{Q}12}=\left(\left(\frac{\varphi_{11}\varphi_{12}}{{\left(1+\left(1-\varphi_{11}^{q}\right)\left(1-\varphi_{12}^{q}\right)\right)}^{\frac{1}{q}}}\right)e^{i2\pi \left(\frac{{Э}_{\varphi_{11}}{Э}_{\varphi_{12}}}{{\left(1+\left(1-{Э}_{\varphi_{11}}^{q}\right)\left(1-{Э}_{\varphi_{12}}^{q}\right)\right)}^{\frac{1}{q}}}\right)},{\left(\frac{\psi_{11}^{q}+\psi_{12}^{q}}{1+\psi_{11}^{q}\psi_{12}^{q}}\right)}^{\frac{1}{q}}e^{i2\pi {\left(\frac{{Э}_{\psi_{11}}^{q}+{Э}_{\psi_{12}}^{q}}{1+{Э}_{\psi_{11}}^{q}{Э}_{\psi_{12}}^{q}}\right)}^{\frac{1}{q}}}\right)$
*3*. $$\eta A_{{}_{Q}11}=\left({\left(\frac{{\left(1+\varphi_{11}^{q}\right)}^{\eta }-{\left(1-\varphi_{11}^{q}\right)}^{\eta }}{{\left(1+\varphi_{11}^{q}\right)}^{\eta }+{\left(1-\varphi_{11}^{q}\right)}^{\eta }}\right)}^{\frac{1}{q}}e^{i2\pi {\left(\frac{{\left(1+{Э}_{\varphi_{11}}^{q}\right)}^{\eta }-{\left(1-{Э}_{\varphi_{11}}^{q}\right)}^{\eta }}{{\left(1+{Э}_{\varphi_{11}}^{q}\right)}^{\eta }+{\left(1-{Э}_{\varphi_{11}}^{q}\right)}^{\eta }}\right)}^{\frac{1}{q}}},\left(\frac{2^{\frac{1}{q}}\psi_{11}^{\eta }}{{\left({\left(2-\psi_{11}^{q}\right)}^{\eta }+{\left(\psi_{11}^{q}\right)}^{\eta }\right)}^{\frac{1}{q}}}\right)e^{i2\pi \left(\frac{2^{\frac{1}{q}}{Э}_{\psi_{11}}^{\eta }}{{\left({\left(2-{Э}_{\psi_{11}}^{q}\right)}^{\eta }+{\left({Э}_{\psi_{11}}^{q}\right)}^{\eta }\right)}^{\frac{1}{q}}}\right)}\right)$$
*4*. $$A_{{}_{Q}11}^{\eta }=\left(\left(\frac{2^{\frac{1}{q}}\varphi_{11}^{\eta }}{{\left({\left(2-\varphi_{11}^{q}\right)}^{\eta }+{\left(\varphi_{11}^{q}\right)}^{\eta }\right)}^{\frac{1}{q}}}\right)e^{i2\pi \left(\frac{2^{\frac{1}{q}}{Э}_{\varphi_{11}}^{\eta }}{{\left({\left(2-{Э}_{\varphi_{11}}^{q}\right)}^{\eta }+{\left({Э}_{\varphi_{11}}^{q}\right)}^{\eta }\right)}^{\frac{1}{q}}}\right)},{\left(\frac{{\left(1+\psi_{11}^{q}\right)}^{\eta }-{\left(1-\psi_{11}^{q}\right)}^{\eta }}{{\left(1+\psi_{11}^{q}\right)}^{\eta }+{\left(1-\psi_{11}^{q}\right)}^{\eta }}\right)}^{\frac{1}{q}}e^{i2\pi {\left(\frac{{\left(1+{Э}_{\psi_{11}}^{q}\right)}^{\eta }-{\left(1-{Э}_{\psi_{11}}^{q}\right)}^{\eta }}{{\left(1+{Э}_{\psi_{11}}^{q}\right)}^{\eta }+{\left(1-{Э}_{\psi_{11}}^{q}\right)}^{\eta }}\right)}^{\frac{1}{q}}}\right)$$

**Theorem** **1.** *For any two Cq-ROFHSNs* $A_{{}_{Q}11}=\left(\varphi_{11}e^{i2\pi {Э}_{\varphi_{11}}},\psi_{11}e^{i2\pi {Э}_{\psi_{11}}}\right)$ *and* $A_{{}_{Q}12}=\left(\varphi_{12}e^{i2\pi {Э}_{\varphi_{12}}},\psi_{12}e^{i2\pi {Э}_{\psi_{12}}}\right)$ *with any* $\eta ,\eta_{1},\eta_{2}>0$ *.*

*1*.
$A_{{}_{Q}11}⊗A_{{}_{Q}12}=A_{{}_{Q}12}⊗A_{{}_{Q}11}$ *;*
*2*.
$A_{{}_{Q}11}⊕A_{{}_{Q}12}=A_{{}_{Q}12}⊕A_{{}_{Q}11}$ *;*
*3*.
$A_{{}_{Q}11}^{\eta }⊗A_{{}_{Q}12}^{\eta }={\left(A_{{}_{Q}11}⊗A_{{}_{Q}12}\right)}^{\eta }$ *;*
*4*.
$\eta A_{{}_{Q}11}⊕\eta A_{{}_{Q}12}=\eta \left(A_{{}_{Q}11}⊕A_{{}_{Q}12}\right)$ *;*
*5*.
$A_{{}_{Q}11}^{\eta_{1}}⊗A_{{}_{Q}11}^{\eta_{2}}=A_{{}_{Q}11}^{\eta_{1}+\eta_{2}}$ *;*
*6*.
$\eta_{1}A_{{}_{Q}11}⊕\eta_{2}A_{{}_{Q}11}=\left(\eta_{1}+\eta_{2}\right)A_{{}_{Q}11}$ *;*

According to the operational laws in Definition 7, we present the Cq-ROFHSEWA and Cq-ROFHSEWG operators in this section. These operators are then defined in order to compensate for their shortcomings in aggregating Cq-ROFHSNs. In other words, Einstein weighted averaging tends to group arguments primarily, whereas Einstein weighted geometric tends to group personal arguments primarily.

### 3.2. Aggregation Operator for Weighted Averaging of Cq-ROFHSNs 

Based on the operational laws in Definition 7, the Cq-ROFHSEWA operator is defined to aggregate Cq-ROFHSNs information.

**Definition** **9.** *Let* $A_{{}_{Q}\mathrm{rt}}=Q_{\overset{⌣}{D}}^{x}=\left(\varphi_{Q\left(d_{t}\right)}\left(x_{r}\right)e^{i2\pi {Э}_{\varphi_{Q\left(d_{t}\right)}\left(x_{r}\right)}},\psi_{Q\left(d_{t}\right)}\left(x_{r}\right)e^{i2\pi {Э}_{\psi_{Q\left(d_{t}\right)}\left(x_{r}\right)}}\right)=\left(\varphi_{\mathrm{rt}}e^{i2\pi {Э}_{\varphi_{\mathrm{rt}}}},\psi_{\mathrm{rt}}e^{i2\pi {Э}_{\psi_{\mathrm{rt}}}}\right)$ *be a Cq-ROFHSN,* ${Ř}_{r}$ *and* ${Ť}_{t}$ *, represent the weights of experts and attributes, respectively, under the following circumstances* ${Ř}_{r}>0$ *,* $\sum_{r=1}^{m}{Ř}_{r}=1$ *,* ${Ť}_{t}>0$ *, and* $\sum_{t=1}^{n}{Ť}_{t}=1$ *. Then, the complex q-rung orthopair fuzzy hypersoft Einstein weighted averaging (Cq-ROFHSEWA) operator is defined as follows:*

$$Cq-ROFHSEWA\left(A_{{}_{Q}11},A_{{}_{Q}12},\ldots ,A_{{}_{Q}\mathrm{rt}}\right)={⊕}_{t=1}^{n}{Ť}_{t}\left({⊕}_{r=1}^{m}{Ř}_{r}A_{{}_{Q}\mathrm{rt}}\right)$$

**Theorem** **2.** *Let* $A_{{}_{Q}\mathrm{rt}}=Q_{\overset{⌣}{D}}^{x}=\left(\varphi_{Q\left(d_{t}\right)}\left(x_{r}\right)e^{i2\pi {Э}_{\varphi_{Q\left(d_{t}\right)}\left(x_{r}\right)}},\psi_{Q\left(d_{t}\right)}\left(x_{r}\right)e^{i2\pi {Э}_{\psi_{Q\left(d_{t}\right)}\left(x_{r}\right)}}\right)=\left(\varphi_{\mathrm{rt}}e^{i2\pi {Э}_{\varphi_{\mathrm{rt}}}},\psi_{\mathrm{rt}}e^{i2\pi {Э}_{\psi_{\mathrm{rt}}}}\right)$ $\left(r=1,2,\ldots ,m,t=1,2,\ldots ,n\right)$ *be a group of Cq-ROFHSNs with the weights of experts and attributes* ${Ř}_{r}$ *and* ${Ť}_{t}$ *for* ${Ř}_{r}>0$ *,* $\sum_{r=1}^{m}{Ř}_{r}=1$ *,* ${Ť}_{t}>0$ *, and* $\sum_{t=1}^{n}{Ť}_{t}=1$ *. Then, the aggregated result of the Cq-ROFHSEWA operator is still Cq-ROFHSN, which is obtained by the equation:*

$$Cq-ROFHSEWA\left(A_{{}_{Q}11},A_{{}_{Q}12},\ldots ,A_{{}_{Q}\mathrm{rt}}\right)={⊕}_{t=1}^{n}{Ť}_{t}\left({⊕}_{r=1}^{m}{Ř}_{r}A_{{}_{Q}\mathrm{rt}}\right)$$

(11) $$=\left(\begin{array}{cc} \; & {\left(\frac{{\prod_{t=1}^{n}\left(\prod_{r=1}^{m}{\left(1+\varphi_{\mathrm{rt}}^{q}\right)}^{{\overset{ˇ}{R}}_{r}}\right)}^{{\overset{ˇ}{T}}_{t}}-{\prod_{t=1}^{n}\left(\prod_{r=1}^{m}{\left(1-\varphi_{\mathrm{rt}}^{q}\right)}^{{\overset{ˇ}{R}}_{r}}\right)}^{{\overset{ˇ}{T}}_{t}}}{{\prod_{t=1}^{n}\left(\prod_{r=1}^{m}{\left(1+\varphi_{\mathrm{rt}}^{q}\right)}^{{\overset{ˇ}{R}}_{r}}\right)}^{{\overset{ˇ}{T}}_{t}}+{\prod_{t=1}^{n}\left(\prod_{r=1}^{m}{\left(1-\varphi_{\mathrm{rt}}^{q}\right)}^{{\overset{ˇ}{R}}_{r}}\right)}^{{\overset{ˇ}{T}}_{t}}}\right)}^{\frac{1}{q}}e^{i2\pi {\left(\frac{{\prod_{t=1}^{n}\left(\prod_{r=1}^{m}{\left(1+{Э}_{\varphi_{\mathrm{rt}}}^{q}\right)}^{{\overset{ˇ}{R}}_{r}}\right)}^{{\overset{ˇ}{T}}_{t}}-{\prod_{t=1}^{n}\left(\prod_{r=1}^{m}{\left(1-{Э}_{\varphi_{\mathrm{rt}}}^{q}\right)}^{{\overset{ˇ}{R}}_{r}}\right)}^{{\overset{ˇ}{T}}_{t}}}{{\prod_{t=1}^{n}\left(\prod_{r=1}^{m}{\left(1+{Э}_{\varphi_{\mathrm{rt}}}^{q}\right)}^{{\overset{ˇ}{R}}_{r}}\right)}^{{\overset{ˇ}{T}}_{t}}+{\prod_{t=1}^{n}\left(\prod_{r=1}^{m}{\left(1-{Э}_{\varphi_{\mathrm{rt}}}^{q}\right)}^{{\overset{ˇ}{R}}_{r}}\right)}^{{\overset{ˇ}{T}}_{t}}}\right)}^{\frac{1}{q}}},\\ \; & \frac{2^{\frac{1}{q}}{\prod_{t=1}^{n}\left(\prod_{r=1}^{m}{\left(\psi_{\mathrm{rt}}\right)}^{{\overset{ˇ}{R}}_{r}}\right)}^{{\overset{ˇ}{T}}_{t}}}{{\left({\prod_{t=1}^{n}\left(\prod_{r=1}^{m}{\left(2-\psi_{\mathrm{rt}}^{q}\right)}^{{\overset{ˇ}{R}}_{r}}\right)}^{{\overset{ˇ}{T}}_{t}}+{\prod_{t=1}^{n}\left(\prod_{r=1}^{m}{\left(\psi_{\mathrm{rt}}^{q}\right)}^{{\overset{ˇ}{R}}_{r}}\right)}^{{\overset{ˇ}{T}}_{t}}\right)}^{\frac{1}{q}}}e^{i2\pi \left(\frac{2^{\frac{1}{q}}{\prod_{t=1}^{n}\left(\prod_{r=1}^{m}{\left({Э}_{\psi_{\mathrm{rt}}}\right)}^{{\overset{ˇ}{R}}_{r}}\right)}^{{\overset{ˇ}{T}}_{t}}}{{\left({\prod_{t=1}^{n}\left(\prod_{r=1}^{m}{\left(2-{Э}_{\psi_{\mathrm{rt}}}^{q}\right)}^{{\overset{ˇ}{R}}_{r}}\right)}^{{\overset{ˇ}{T}}_{t}}+{\prod_{t=1}^{n}\left(\prod_{r=1}^{m}{\left({Э}_{\psi_{\mathrm{rt}}}^{q}\right)}^{{\overset{ˇ}{R}}_{r}}\right)}^{{\overset{ˇ}{T}}_{t}}\right)}^{\frac{1}{q}}}\right)} \end{array}\right)$$

The proof of Theorem 2 is presented in the “Appendix A”.

Now, we state some basic properties of the proposed Cq-ROFHSEWA operator in the following Theorem.

**Theorem** **3.** *The Cq-ROFHSEWA operator implies these properties:*

*(1)*  *(Idempotency) If all* $A_{{}_{Q}\mathrm{rt}}$ $\left(r=1,2,\ldots ,m,t=1,2,\ldots ,n\right)$ *are equal, i.e.,* $A_{{}_{Q}\mathrm{rt}}=A_{{}_{Q}}$ *for all* r and t *, then* $Cq-ROFHSEWA\left(A_{{}_{Q}11},A_{{}_{Q}12},\ldots ,A_{{}_{Q}\mathrm{rt}}\right)=A_{{}_{Q}}$ *.*
*(2)*  *(Boundedness) Let*$A_{{}_{Q}\mathrm{rt}}$$\left(r=1,2,\ldots ,m,t=1,2,\ldots ,n\right)$*be a collection of Cq-ROFHSNs, and let*$A_{{}_{Q}}^{-}=\left(\min\varphi_{\mathrm{rt}}e^{i2\pi \min{Э}_{\varphi_{\mathrm{rt}}}},\max\psi_{\mathrm{rt}}e^{i2\pi \max{Э}_{\psi_{\mathrm{rt}}}}\right)$*,*$A_{{}_{Q}}^{+}=\left(\max\varphi_{\mathrm{rt}}e^{i2\pi \max{Э}_{\varphi_{\mathrm{rt}}}},\min\psi_{\mathrm{rt}}e^{i2\pi \min{Э}_{\psi_{\mathrm{rt}}}}\right)$*, then*$A_{{}_{Q}}^{-}\subseteq Cq-ROFHSEWA\left(A_{{}_{Q}11},A_{{}_{Q}12},\ldots ,A_{{}_{Q}\mathrm{rt}}\right)\subseteq A_{{}_{Q}}^{+}$.
*(3)*  *(Monotonicity) Let* $A_{{}_{Q}\mathrm{rt}}$ $\left(r=1,2,\ldots ,m,t=1,2,\ldots ,n\right)$ *and* ${\ddot{A}}_{{}_{Q}\mathrm{rt}}$ $\left(r=1,2,\ldots ,m,t=1,2,\ldots ,n\right)$ *be two sets of Cq-ROFHSNs, if* $A_{{}_{Q}\mathrm{rt}}\subseteq {\ddot{A}}_{{}_{Q}\mathrm{rt}}$ *for all* r and t *, then* $Cq-ROFHSEWA\left(A_{{}_{Q}11},A_{{}_{Q}12},\ldots ,A_{{}_{Q}\mathrm{rt}}\right)\subseteq Cq-ROFHSEWA\left({\ddot{A}}_{{}_{Q}11},{\ddot{A}}_{{}_{Q}12},\ldots ,{\ddot{A}}_{{}_{Q}\mathrm{rt}}\right)$ *.*

Further, we give a complex q-rung orthopair fuzzy hypersoft Einstein weighted geometric averaging (Cq-ROFHSEWG) operator below:

### 3.3. Geometric Aggregator of Cq-ROFHSNs with Weighted Geometric Averaging 

In this section, geometric aggregation operators based on Einstein operations are discussed. We propose a complex q-rung orthopair fuzzy hypersoft Einstein weighted geometric average (Cq-ROFHSEWG) operator based on the Einstein operation proposed in Definition 7. The proposed Cq-ROFHSEWG operator is validated using the induction method. Additionally, the Cq-ROFHSEWG operator is examined in terms of some other properties.

**Definition** **10.** *Consider* $A_{{}_{Q}\mathrm{rt}}=Q_{\overset{⌣}{D}}^{x}=\left(\varphi_{Q\left(d_{t}\right)}\left(x_{r}\right)e^{i2\pi {Э}_{\varphi_{Q\left(d_{t}\right)}\left(x_{r}\right)}},\psi_{Q\left(d_{t}\right)}\left(x_{r}\right)e^{i2\pi {Э}_{\psi_{Q\left(d_{t}\right)}\left(x_{r}\right)}}\right)=\left(\varphi_{\mathrm{rt}}e^{i2\pi {Э}_{\varphi_{\mathrm{rt}}}},\psi_{\mathrm{rt}}e^{i2\pi {Э}_{\psi_{\mathrm{rt}}}}\right)$ *as a collection of Cq-ROFHSNs. Therefore, the Cq-ROFHSEWG operator is a map ∆^n^ →∆, as shown in the following diagram*

$$Cq-ROFHSEWG\left(A_{{}_{Q}11},A_{{}_{Q}12},\ldots ,A_{{}_{Q}\mathrm{rt}}\right)={⊗}_{ΐ=1}^{m}{\left({⊗}_{ѓ=1}^{n}A_{{}_{Q}\mathrm{rt}}{}^{{Ř}_{r}}\right)}^{{Ť}_{t}}$$

*where* ${Ř}_{r}$ *and* ${Ť}_{t}$ *represent the weights of experts and attributes, respectively, under the following circumstances* ${Ř}_{r}>0$ *,* $\sum_{r=1}^{m}{Ř}_{r}=1$ *,* ${Ť}_{t}>0$ *, and* $\sum_{t=1}^{n}{Ť}_{t}=1$ *.*

**Theorem** **4.** *Let* $A_{{}_{Q}\mathrm{rt}}=Q_{\overset{⌣}{D}}^{x}=\left(\varphi_{Q\left(d_{t}\right)}\left(x_{r}\right)e^{i2\pi {Э}_{\varphi_{Q\left(d_{t}\right)}\left(x_{r}\right)}},\psi_{Q\left(d_{t}\right)}\left(x_{r}\right)e^{i2\pi {Э}_{\psi_{Q\left(d_{t}\right)}\left(x_{r}\right)}}\right)=\left(\varphi_{\mathrm{rt}}e^{i2\pi {Э}_{\varphi_{rt}}},\psi_{rt}e^{i2\pi {Э}_{\psi_{rt}}}\right)$ $\left(r=1,2,\ldots ,m,t=1,2,\ldots ,n\right)$ *be a group of Cq-ROFHSNs with the weights of experts and attributes* ${Ř}_{r}$ *and* ${Ť}_{t}$ *for* ${Ř}_{r}>0$ *,* $\sum_{r=1}^{m}{Ř}_{r}=1$ *,* ${Ť}_{t}>0$ *, and* $\sum_{t=1}^{n}{Ť}_{t}=1$ *. Then, the aggregated result of the Cq-ROFHSEWG operator is still Cq-ROFHSN, which is obtained by the equation:*

$$Cq-ROFHSEWG\left(A_{{}_{Q}11},A_{{}_{Q}12},\ldots ,A_{{}_{Q}rt}\right)={⊗}_{ΐ=1}^{m}{\left({⊗}_{ѓ=1}^{n}A_{{}_{Q}rt}{}^{{Ř}_{r}}\right)}^{{Ť}_{t}}\;$$

(12) $$=\left(\begin{array}{cc} \; & \frac{2^{\frac{1}{q}}{\prod_{t=1}^{n}\left(\prod_{r=1}^{m}{\left(\varphi_{rt}\right)}^{{\overset{ˇ}{R}}_{r}}\right)}^{{\overset{ˇ}{T}}_{t}}}{{\left({\prod_{t=1}^{n}\left(\prod_{r=1}^{m}{\left(2-\varphi_{rt}^{q}\right)}^{{\overset{ˇ}{R}}_{r}}\right)}^{{\overset{ˇ}{T}}_{t}}+{\prod_{t=1}^{n}\left(\prod_{r=1}^{m}{\left(\varphi_{rt}^{q}\right)}^{{\overset{ˇ}{R}}_{r}}\right)}^{{\overset{ˇ}{T}}_{t}}\right)}^{\frac{1}{q}}}e^{i2\pi \left(\frac{2^{\frac{1}{q}}{\prod_{t=1}^{n}\left(\prod_{r=1}^{m}{\left({Э}_{\varphi_{rt}}\right)}^{{\overset{ˇ}{R}}_{r}}\right)}^{{\overset{ˇ}{T}}_{t}}}{{\left({\prod_{t=1}^{n}\left(\prod_{r=1}^{m}{\left(2-{Э}_{\varphi_{rt}}^{q}\right)}^{{\overset{ˇ}{R}}_{r}}\right)}^{{\overset{ˇ}{T}}_{t}}+{\prod_{t=1}^{n}\left(\prod_{r=1}^{m}{\left({Э}_{\varphi_{rt}}^{q}\right)}^{{\overset{ˇ}{R}}_{r}}\right)}^{{\overset{ˇ}{T}}_{t}}\right)}^{\frac{1}{q}}}\right)},\\ \; & {\left(\frac{{\prod_{t=1}^{n}\left(\prod_{r=1}^{m}{\left(1+\psi_{rt}^{q}\right)}^{{\overset{ˇ}{R}}_{r}}\right)}^{{\overset{ˇ}{T}}_{t}}-{\prod_{t=1}^{n}\left(\prod_{r=1}^{m}{\left(1-\psi_{rt}^{q}\right)}^{{\overset{ˇ}{R}}_{r}}\right)}^{{\overset{ˇ}{T}}_{t}}}{{\prod_{t=1}^{n}\left(\prod_{r=1}^{m}{\left(1+\psi_{rt}^{q}\right)}^{{\overset{ˇ}{R}}_{r}}\right)}^{{\overset{ˇ}{T}}_{t}}+{\prod_{t=1}^{n}\left(\prod_{r=1}^{m}{\left(1-\psi_{rt}^{q}\right)}^{{\overset{ˇ}{R}}_{r}}\right)}^{{\overset{ˇ}{T}}_{t}}}\right)}^{\frac{1}{q}}e^{i2\pi {\left(\frac{{\prod_{t=1}^{n}\left(\prod_{r=1}^{m}{\left(1+{Э}_{\psi_{rt}}^{q}\right)}^{{\overset{ˇ}{R}}_{r}}\right)}^{{\overset{ˇ}{T}}_{t}}-{\prod_{t=1}^{n}\left(\prod_{r=1}^{m}{\left(1-{Э}_{\psi_{rt}}^{q}\right)}^{{\overset{ˇ}{R}}_{r}}\right)}^{{\overset{ˇ}{T}}_{t}}}{{\prod_{t=1}^{n}\left(\prod_{r=1}^{m}{\left(1+{Э}_{\psi_{rt}}^{q}\right)}^{{\overset{ˇ}{R}}_{r}}\right)}^{{\overset{ˇ}{T}}_{t}}+{\prod_{t=1}^{n}\left(\prod_{r=1}^{m}{\left(1-{Э}_{\psi_{rt}}^{q}\right)}^{{\overset{ˇ}{R}}_{r}}\right)}^{{\overset{ˇ}{T}}_{t}}}\right)}^{\frac{1}{q}}} \end{array}\right)$$

**Proof** **of** **Theorem 4.** Similarly to Theorem 2, Theorem 4 can easily be proved, which is omitted here.

Now, we state some basic properties of the proposed Cq-ROFHSEWG operator in the following Theorem.

**Theorem** **5.** *The Cq-ROFHSEWG operator implies these properties:*

*(1)*  *(Idempotency) If all*$A_{{}_{Q}rt}$$\left(r=1,2,\ldots ,m,t=1,2,\ldots ,n\right)$*are equal, i.e.,*$A_{{}_{Q}rt}=A_{{}_{Q}}$*for all*r and t*, then*$Cq-ROFHSEWG\left(A_{{}_{Q}11},A_{{}_{Q}12},\ldots ,A_{{}_{Q}rt}\right)=A_{{}_{Q}}$.
*(2)*  *(Boundedness) Let*$A_{{}_{Q}rt}$$\left(r=1,2,\ldots ,m,t=1,2,\ldots ,n\right)$*be a collection of Cq-ROFHSNs, and let*$A_{{}_{Q}}^{-}=\left(\min\varphi_{rt}e^{i2\pi \min{Э}_{\varphi_{rt}}},\max\psi_{rt}e^{i2\pi \max{Э}_{\psi_{rt}}}\right)$*,*$A_{{}_{Q}}^{+}=\left(\max\varphi_{rt}e^{i2\pi \max{Э}_{\varphi_{rt}}},\min\psi_{rt}e^{i2\pi \min{Э}_{\psi_{rt}}}\right)$*, then*$A_{{}_{Q}}^{-}\subseteq Cq-ROFHSEWG\left(A_{{}_{Q}11},A_{{}_{Q}12},\ldots ,A_{{}_{Q}rt}\right)\subseteq A_{{}_{Q}}^{+}$.

*(Monotonicity) Let*$A_{{}_{Q}rt}$$\left(r=1,2,\ldots ,m,t=1,2,\ldots ,n\right)$*and*${\ddot{A}}_{{}_{Q}rt}$$\left(r=1,2,\ldots ,m,t=1,2,\ldots ,n\right)$*be two sets of Cq-ROFHSNs; if*$A_{{}_{Q}rt}\subseteq {\ddot{A}}_{{}_{Q}rt}$*for all*r and t*, then*$Cq-ROFHSEWG\left(A_{{}_{Q}11},A_{{}_{Q}12},\ldots ,A_{{}_{Q}rt}\right)\subseteq Cq-ROFHSEWG\left({\ddot{A}}_{{}_{Q}11},{\ddot{A}}_{{}_{Q}12},\ldots ,{\ddot{A}}_{{}_{Q}rt}\right)$.

**Proof** **of** **Theorem 5.** This can be proved analogously.

## 4. An Approach to Multi-Attribute Decision Making under Cq-ROFHSS Environments

### 4.1. Proposed Approach to Solve the MADM Problem

We present an application based on a complex q-rung orthopair fuzzy Einstein operator to solve the MADM problem in this section. Consider $\left\{\left.Y^{\left(\varsigma \right)}\right|\varsigma =1,2,\ldots ,z\right\}$ to be a set of s alternatives and $U=\{u_{1},u_{2},\ldots ,u_{n}\}$ to be a set of n experts. The weights of experts are given as $ℭ=\{{ℭ}_{1},{ℭ}_{2},\ldots ,{ℭ}_{n}\}$ and ${ℭ}_{i}>0$, $\sum_{i=1}^{n}{ℭ}_{i}=1$. Let $V=\{V_{1},V_{2},\ldots ,V_{m}\}$ be a set of attributes with their corresponding multi sub-attributes such as $\tilde{V}=\{V_{1\tau },V_{2\tau },\ldots ,V_{m\tau }\}$ for all $\tau \in \left\{1,2,\ldots ,t\right\}$ with weights $\varepsilon =\{\varepsilon_{1\tau },\varepsilon_{2\tau },\ldots ,\varepsilon_{m\tau }\}$, such as $\varepsilon_{\tau }>0$, $\sum_{\tau =1}^{t}\varepsilon_{\tau }=1$. The components in the collection of sub-attributes are multi-valued; for the sake of accessibility, the components of $\tilde{V}$ can be stated as $\tilde{V}=\left\{Q_{ƛ}:ƛ\in \left\{1,2,\ldots ,k\right\}\right\}$. The team of experts $U=\{u_{1},u_{2},\ldots ,u_{n}\}$ appraise the alternatives $\left\{\left.Y^{\left(\varsigma \right)}\right|\varsigma =1,2,\ldots ,z\right\}$ under the preferred sub-attributes of the considered parameters $Q_{ƛ}:ƛ\in \left\{1,2,\ldots ,k\right\}$ given in the form of Cq-ROFHSNs such as ${\left(A_{{}_{Q}rt}^{\left(\varsigma \right)}\right)}_{n\times ƛ}=\left(\varphi_{rt}^{\left(\varsigma \right)}e^{i2\pi {Э}_{\varphi_{rt}}^{\left(\varsigma \right)}},\psi_{rt}^{\left(\varsigma \right)}e^{i2\pi {Э}_{\psi_{rt}}^{\left(\varsigma \right)}}\right)$ where $0\leq \varphi_{rt}^{\left(\varsigma \right)},\psi_{rt}^{\left(\varsigma \right)},{Э}_{\varphi_{rt}}^{\left(\varsigma \right)},{Э}_{\psi_{rt}}^{\left(\varsigma \right)}\leq 1$ and $0\leq \varphi_{rt}^{\left(\varsigma \right)}+\psi_{rt}^{\left(\varsigma \right)}\leq 1,0\leq {Э}_{\varphi_{rt}}^{\left(\varsigma \right)}+{Э}_{\psi_{rt}}^{\left(\varsigma \right)}\leq 1,$ for all r,t. 

The normalization of the decision matrix is required. By adding criteria such as cost to the decision matrix, we standardize the decision matrix when there are different types of criteria or attributes, such as cost and benefit. The usual complex q-rung orthopair fuzzy hypersoft decision matrix is obtained. It assesses the resulting matrix A using two types of attributes, namely a benefit attribute and a cost attribute. The ${\left(A_{{}_{Q}rt}^{\left(\varsigma \right)}\right)}_{n\times ƛ}$ normalization formula may be used to standardize the performance rating matrix ${\left(A_{{}_{Q}rt}^{\left(\varsigma \right)}\right)}_{n\times ƛ}$ into a normalized matrix ${\left({\hat{A}}_{{}_{Q}rt}^{\left(\varsigma \right)}\right)}_{n\times ƛ}$ if all the attributes in the MADM are of the same type, whereas if all the attributes are of the same type, a normalization formula may be used. Now, by utilizing the proposed weighted aggregation operators, we develop an algorithm to solve the MADM under Cq-ROFHS environment which is given in Algorithm 1.

Following is an algorithm based on Cq-ROFHSSs for selecting the most appropriate option (see Algorithm 1).
**Algorithm 1:** Selection of a suitable object using Cq-ROFHSS**Input:**(i)$\left\{\left.Y^{\left(\varsigma \right)}\right|\varsigma =1,2,\ldots ,z\right\}$, a universal set of n alternatives, $U=\{u_{1},u_{2},\ldots ,u_{n}\}$ to be a set of n experts,(ii)A Cq-ROFHSS ${\left(A_{{}_{Q}rt}^{\left(\varsigma \right)}\right)}_{n\times ƛ,}$ where a complex q-rung orthopair fuzzy hypersoft decision matrix is provided by ${\left(A_{{}_{Q}rt}^{\left(\varsigma \right)}\right)}_{n\times ƛ}=\left(\varphi_{rt}^{\left(\varsigma \right)}e^{i2\pi {Э}_{\varphi_{rt}}^{\left(\varsigma \right)}},\psi_{rt}^{\left(\varsigma \right)}e^{i2\pi {Э}_{\psi_{rt}}^{\left(\varsigma \right)}}\right)$ in a tabular format,(iii)The weights of experts and attributes ${Ř}_{r}$ and ${Ť}_{t}$ for ${Ř}_{r}>0$, $\sum_{r=1}^{m}{Ř}_{r}=1$, ${Ť}_{t}>0$, and $\sum_{t=1}^{n}{Ť}_{t}=1$. **Output**: The object having maximum final score value will be the decision object. **begin** 1.**for**ς = 1 to z **do**2.  **for** r = 1 to m **do**3.   **for** t = 1 to n **do**4.Aggregate the Cq-ROFHSN for each alternative $\left\{\left.Y^{\left(\varsigma \right)}\right|\varsigma =1,2,\ldots ,z\right\}$ by using the Cq-ROFHSEWA operator,$Cq-ROFHSEWA\left(A_{{}_{Q}11}^{\left(\varsigma \right)},A_{{}_{Q}12}^{\left(\varsigma \right)},\ldots ,A_{{}_{Q}rt}^{\left(\varsigma \right)}\right)={⊕}_{t=1}^{n}{Ť}_{t}\left({⊕}_{r=1}^{m}{Ř}_{r}A_{{}_{Q}rt}^{\left(\varsigma \right)}\right)$$=\left(\begin{array}{cc} \; & {\left(\frac{{\prod_{t=1}^{n}\left(\prod_{r=1}^{m}{\left(1+{\left(\varphi_{rt}^{\left(\varsigma \right)}\right)}^{q}\right)}^{{\overset{ˇ}{R}}_{r}}\right)}^{{\overset{ˇ}{T}}_{t}}-{\prod_{t=1}^{n}\left(\prod_{r=1}^{m}{\left(1-{\left(\varphi_{rt}^{\left(\varsigma \right)}\right)}^{q}\right)}^{{\overset{ˇ}{R}}_{r}}\right)}^{{\overset{ˇ}{T}}_{t}}}{{\prod_{t=1}^{n}\left(\prod_{r=1}^{m}{\left(1+{\left(\varphi_{rt}^{\left(\varsigma \right)}\right)}^{q}\right)}^{{\overset{ˇ}{R}}_{r}}\right)}^{{\overset{ˇ}{T}}_{t}}+{\prod_{t=1}^{n}\left(\prod_{r=1}^{m}{\left(1-{\left(\varphi_{rt}^{\left(\varsigma \right)}\right)}^{q}\right)}^{{\overset{ˇ}{R}}_{r}}\right)}^{{\overset{ˇ}{T}}_{t}}}\right)}^{\frac{1}{q}}e^{i2\pi {\left(\frac{{\prod_{t=1}^{n}\left(\prod_{r=1}^{m}{\left(1+{\left({Э}_{\varphi_{rt}}^{\left(\varsigma \right)}\right)}^{q}\right)}^{{\overset{ˇ}{R}}_{r}}\right)}^{{\overset{ˇ}{T}}_{t}}-{\prod_{t=1}^{n}\left(\prod_{r=1}^{m}{\left(1-{\left({Э}_{\varphi_{rt}}^{\left(\varsigma \right)}\right)}^{q}\right)}^{{\overset{ˇ}{R}}_{r}}\right)}^{{\overset{ˇ}{T}}_{t}}}{{\prod_{t=1}^{n}\left(\prod_{r=1}^{m}{\left(1+{\left({Э}_{\varphi_{rt}}^{\left(\varsigma \right)}\right)}^{q}\right)}^{{\overset{ˇ}{R}}_{r}}\right)}^{{\overset{ˇ}{T}}_{t}}+{\prod_{t=1}^{n}\left(\prod_{r=1}^{m}{\left(1-{\left({Э}_{\varphi_{rt}}^{\left(\varsigma \right)}\right)}^{q}\right)}^{{\overset{ˇ}{R}}_{r}}\right)}^{{\overset{ˇ}{T}}_{t}}}\right)}^{\frac{1}{q}}},\\ \; & \frac{2^{\frac{1}{q}}{\prod_{t=1}^{n}\left(\prod_{r=1}^{m}{\left(\psi_{rt}^{\left(\varsigma \right)}\right)}^{{\overset{ˇ}{R}}_{r}}\right)}^{{\overset{ˇ}{T}}_{t}}}{{\left({\prod_{t=1}^{n}\left(\prod_{r=1}^{m}{\left(2-{\left(\psi_{rt}^{\left(\varsigma \right)}\right)}^{q}\right)}^{{\overset{ˇ}{R}}_{r}}\right)}^{{\overset{ˇ}{T}}_{t}}+{\prod_{t=1}^{n}\left(\prod_{r=1}^{m}{\left({\left(\psi_{rt}^{\left(\varsigma \right)}\right)}^{q}\right)}^{{\overset{ˇ}{R}}_{r}}\right)}^{{\overset{ˇ}{T}}_{t}}\right)}^{\frac{1}{q}}}e^{i2\pi \left(\frac{2^{\frac{1}{q}}{\prod_{t=1}^{n}\left(\prod_{r=1}^{m}{\left({Э}_{\psi_{rt}}^{\left(\varsigma \right)}\right)}^{{\overset{ˇ}{R}}_{r}}\right)}^{{\overset{ˇ}{T}}_{t}}}{{\left({\prod_{t=1}^{n}\left(\prod_{r=1}^{m}{\left(2-{\left({Э}_{\psi_{rt}}^{\left(\varsigma \right)}\right)}^{q}\right)}^{{\overset{ˇ}{R}}_{r}}\right)}^{{\overset{ˇ}{T}}_{t}}+{\prod_{t=1}^{n}\left(\prod_{r=1}^{m}{\left({\left({Э}_{\psi_{rt}}^{\left(\varsigma \right)}\right)}^{q}\right)}^{{\overset{ˇ}{R}}_{r}}\right)}^{{\overset{ˇ}{T}}_{t}}\right)}^{\frac{1}{q}}}\right)} \end{array}\right)$or by a utilizing Cq-ROFHSEWG operator.$Cq-ROFHSEWG\left(A_{{}_{Q}11},A_{{}_{Q}12},\ldots ,A_{{}_{Q}rt}\right)={⊗}_{ΐ=1}^{m}{\left({⊗}_{ѓ=1}^{n}A_{{}_{Q}rt}{}^{{Ř}_{r}}\right)}^{{Ť}_{t}}$$=\left(\begin{array}{cc} \; & \frac{2^{\frac{1}{q}}{\prod_{t=1}^{n}\left(\prod_{r=1}^{m}{\left(\varphi_{rt}^{\left(\varsigma \right)}\right)}^{{\overset{ˇ}{R}}_{r}}\right)}^{{\overset{ˇ}{T}}_{t}}}{{\left({\prod_{t=1}^{n}\left(\prod_{r=1}^{m}{\left(2-{\left(\varphi_{rt}^{\left(\varsigma \right)}\right)}^{q}\right)}^{{\overset{ˇ}{R}}_{r}}\right)}^{{\overset{ˇ}{T}}_{t}}+{\prod_{t=1}^{n}\left(\prod_{r=1}^{m}{\left({\left(\varphi_{rt}^{\left(\varsigma \right)}\right)}^{q}\right)}^{{\overset{ˇ}{R}}_{r}}\right)}^{{\overset{ˇ}{T}}_{t}}\right)}^{\frac{1}{q}}}e^{i2\pi \left(\frac{2^{\frac{1}{q}}{\prod_{t=1}^{n}\left(\prod_{r=1}^{m}{\left({Э}_{\varphi_{rt}}^{\left(\varsigma \right)}\right)}^{{\overset{ˇ}{R}}_{r}}\right)}^{{\overset{ˇ}{T}}_{t}}}{{\left({\prod_{t=1}^{n}\left(\prod_{r=1}^{m}{\left(2-{\left({Э}_{\varphi_{rt}}^{\left(\varsigma \right)}\right)}^{q}\right)}^{{\overset{ˇ}{R}}_{r}}\right)}^{{\overset{ˇ}{T}}_{t}}+{\prod_{t=1}^{n}\left(\prod_{r=1}^{m}{\left({\left({Э}_{\varphi_{rt}}^{\left(\varsigma \right)}\right)}^{q}\right)}^{{\overset{ˇ}{R}}_{r}}\right)}^{{\overset{ˇ}{T}}_{t}}\right)}^{\frac{1}{q}}}\right)},\\ \; & {\left(\frac{{\prod_{t=1}^{n}\left(\prod_{r=1}^{m}{\left(1+{\left(\psi_{rt}^{\left(\varsigma \right)}\right)}^{q}\right)}^{{\overset{ˇ}{R}}_{r}}\right)}^{{\overset{ˇ}{T}}_{t}}-{\prod_{t=1}^{n}\left(\prod_{r=1}^{m}{\left(1-{\left(\psi_{rt}^{\left(\varsigma \right)}\right)}^{q}\right)}^{{\overset{ˇ}{R}}_{r}}\right)}^{{\overset{ˇ}{T}}_{t}}}{{\prod_{t=1}^{n}\left(\prod_{r=1}^{m}{\left(1+{\left(\psi_{rt}^{\left(\varsigma \right)}\right)}^{q}\right)}^{{\overset{ˇ}{R}}_{r}}\right)}^{{\overset{ˇ}{T}}_{t}}+{\prod_{t=1}^{n}\left(\prod_{r=1}^{m}{\left(1-{\left(\psi_{rt}^{\left(\varsigma \right)}\right)}^{q}\right)}^{{\overset{ˇ}{R}}_{r}}\right)}^{{\overset{ˇ}{T}}_{t}}}\right)}^{\frac{1}{q}}e^{i2\pi {\left(\frac{{\prod_{t=1}^{n}\left(\prod_{r=1}^{m}{\left(1+{\left({Э}_{\psi_{rt}}^{\left(\varsigma \right)}\right)}^{q}\right)}^{{\overset{ˇ}{R}}_{r}}\right)}^{{\overset{ˇ}{T}}_{t}}-{\prod_{t=1}^{n}\left(\prod_{r=1}^{m}{\left(1-{\left({Э}_{\psi_{rt}}^{\left(\varsigma \right)}\right)}^{q}\right)}^{{\overset{ˇ}{R}}_{r}}\right)}^{{\overset{ˇ}{T}}_{t}}}{{\prod_{t=1}^{n}\left(\prod_{r=1}^{m}{\left(1+{\left({Э}_{\psi_{rt}}^{\left(\varsigma \right)}\right)}^{q}\right)}^{{\overset{ˇ}{R}}_{r}}\right)}^{{\overset{ˇ}{T}}_{t}}+{\prod_{t=1}^{n}\left(\prod_{r=1}^{m}{\left(1-{\left({Э}_{\psi_{rt}}^{\left(\varsigma \right)}\right)}^{q}\right)}^{{\overset{ˇ}{R}}_{r}}\right)}^{{\overset{ˇ}{T}}_{t}}}\right)}^{\frac{1}{q}}} \end{array}\right)$5.  **end for**6.**end for**7.**for**ς = 1 to z **do**8.Determine the score functions $Ş\left({\hat{A}}_{{}_{Q}rt}^{\left(\varsigma \right)}\right)$ of every alternative $\left\{Y^{\left(\varsigma \right)}\right\}$ via Equation (7);9.**end for**10.**for**ς = 1 to z **do**11.Calculate final scores for each object by $\max\left\{Ş\left({\hat{A}}_{{}_{Q}rt}^{\left(\varsigma \right)}\right)\right\}$;12.**end for****end**

**Figure 3:**
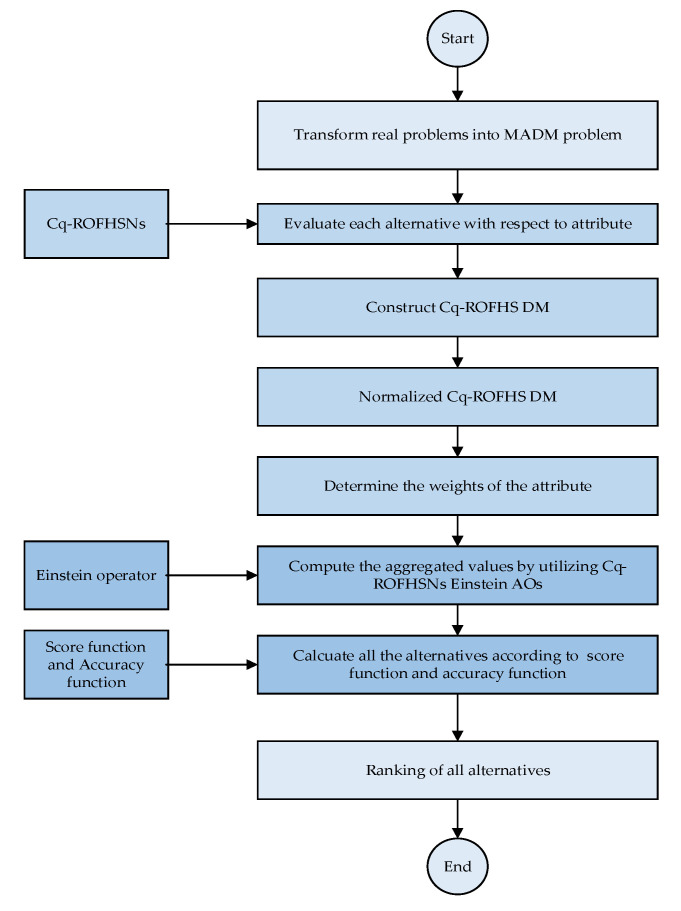
Flowchart of the proposed Method based on Cq-ROFHSSs.

A flowchart of the proposed algorithm is presented in Figure 3.

### 4.2. Numerical Example

**Figure 4:**
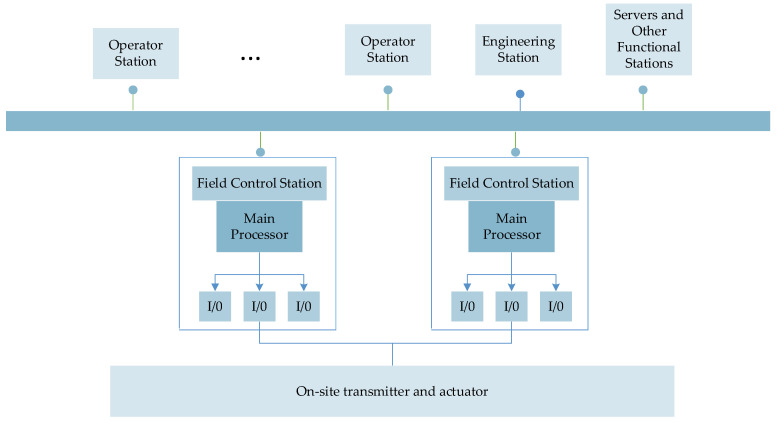
Composition of DCS.

The Distributed Control System (DCS) is a multi-level computer system composed of a process control level and a process monitoring level with a communication network as the link, which integrates 4C technologies such as computer, communication and control, etc. [43,44]. Its basic idea is centralized management and decentralized control. The composition of the Distributed Control System is presented in Figure 4.

As the economy continues to develop, the level of automation technology in chemical enterprises is gradually rising and the scale of production is expanding. In the chemical industry, the input and use requirements of raw materials usually require very strict control of conditions due to special production conditions. DCS is an automation system controlled by a computer. With the emergence of DCS, major chemical enterprises have applied the system in the production process, the system can not only improve the quality of chemical production products but also the accuracy of the production process control, and also the use of a unified management form to monitor and manage the chemical process, more chemical enterprises to reduce production costs [45,46,47]. In the actual production process of chemical enterprises, the integration of the system also increases the market competitiveness for enterprises. The application of the DCS automatic control system in the chemical industry not only improves the interests of chemical enterprises to a certain extent but also promotes the development of the chemical industry.

**Example** **4.** *In order to better manage production, a chemical company wishes to purchase an independent and controllable DCS. There are five different types of DCSs available. It is the responsibility of an expert team hired by the department to evaluate these five sets of DCSs in order to select a system that offers the highest comprehensive performance. There are five different types of DCSs* $Y=\left\{Y^{\left(1\right)},Y^{\left(2\right)},Y^{\left(3\right)},Y^{\left(4\right)},Y^{\left(5\right)}\right\}$ *available for selection. In order to select a system with the highest comprehensive performance from these five types of DCSs, a group of experts hired by the department evaluates these five types of DCSs. To evaluate the alternatives, we consider a set of attributes* $F=\left\{f_{1},f_{2},f_{3},f_{4}\right\}$ *given as f_1_ = Stability; f_2_ = Real-time; f_3_ = Anti-jamming; f_4_ = Applicability of special function blocks. Let the corresponding sub-attribute be given as:*

***Stability** = f_1_ =* $\begin{array}{l} \{f_{11}=Controller\;processing\;cycle\;and\;its\;stability,\\ f_{12}=Controller\;switching\;stability,\\ f_{13}=Network\;switching\;stability\} \end{array}$ *;*
***Real-time** = f_2_ =* $\begin{array}{l} \{f_{21}=switch\;acquisition\;real-time,\\ f_{22}=analog\;acquisition\;real-time,\\ f_{23}=network\;transmission\;real-time\} \end{array}$ *;*
***Anti-jamming*** $=f_{3}=\left\{f_{31}=Anti-jamming\right\}$ *;*
***Applicability*** $=f_{4}=\left\{f_{41}=Applicability\right\}$ *.*

*Let* $F^{\prime }=f_{1}\times f_{2}\times f_{3}\times f_{4}$ *be a set of sub-attributes* $F^{\prime }=f_{1}\times f_{2}\times f_{3}\times f_{4}=\{c_{11},c_{12},c_{13}\}\times \{c_{21},c_{22},c_{23}\}\times \{c_{31}\}\times \{c_{41}\}=\left\{\begin{array}{l} \left\{f_{11},f_{21},f_{31},f_{41}\}\times \{f_{11},f_{22},f_{31},f_{41}\}\times \{f_{11},f_{23},f_{31},f_{41}\}\times \{f_{12},f_{21},f_{31},f_{41}\}\times \{f_{12},f_{22},f_{31},f_{41}\right\}\\ \times \{f_{12},f_{23},f_{31},f_{41}\}\times \{f_{13},f_{21},f_{31},f_{41}\}\times \{f_{13},f_{22},f_{31},f_{41}\}\times \{f_{13},f_{23},f_{31},f_{41}\} \end{array}\right\}$ *, where* $F^{\prime }=\left\{{\dot{f}}_{1},{\dot{f}}_{2},{\dot{f}}_{3},{\dot{f}}_{4},{\dot{f}}_{5},{\dot{f}}_{6},{\dot{f}}_{7},{\dot{f}}_{8},{\dot{f}}_{9}\right\}$ *is a set of all multi sub-attributes with weights* $(0.09,0.07,0.13,0.1,0.06,0.12,0.06,0.19,0.18{)}^{\prime }$ *.*

*Let* $\left\{u_{1},u_{2},u_{3}\right\}$ *be a set of three experts with weights* $(0.19,0.5,0.31{)}^{\prime }$ *to judge the optimum alternative.*

A DCS is also available which is based not only on the overall average rating of an alternative, but also on the most recent reviews. Due to the fact that the average is built on expert reviews over time, it may not always reflect the views of current experts. The most recent reviews regarding the latest version are more likely to reflect current opinions. 

A selection should be chosen based on the overall rating and the most recent version of the alternative. In terms of amplitude and phase, this can be represented using Cq-ROFHSNs expressions. Specialists then provide their preferences in the form of Cq-ROFHSNs. As an example, if the average satisfaction level of expert $u_{1}$ with regard to attribute ${\dot{f}}_{1}$ in alternative Y^(1)^ is 0.36, and the current satisfaction level is 0.74, the Mem can be expressed as 0.36e^i2π0.74^. The NMem, however, indicates the level of dissatisfaction.

For each alternative, DMs will evaluate the ratings in the form of Cq-ROFHSNs under each of the multiple sub-attributes. In order to find the most suitable alternative, the following method has been developed.

**Step 1.** In Table 1, Table 2 and Table 3, experts summarize their priorities and scores in the form of Cq-ROFHSNs.

**Step 2.** There is no need to normalize since all attributes are of the same type.

**Step 3.** Assuming q=1, integrate the attribute information of each distributed control system using the the Cq-ROFHSEWA or Cq-ROFHSEWG operator to obtain the comprehensive attribute information for each distributed control system. The results can be found in Table 4 and Table 5.

**Step 4.** Using the Cq-ROFHSEWA operator, compute the corresponding score function values for each distributed control system:

Ş^(1)^ = 0.1860, Ş^(2)^ = 0.2175, Ş^(3)^ = 0.1622, Ş^(4)^ = 0.2229, and Ş^(5)^ = 0.2476,

Or calculate the similarity of each alternative from the Cq-ROFHSEWG operator:

Ş^(1)^ = 0.7806, Ş^(2)^ = 0.8170, Ş^(3)^ = 0.7831, Ş^(4)^ = 0.8210, or Ş^(5)^= 0.8412.

**Step 5.** Based on the score function value, rank the advantages and disadvantages of the five Distributed Control Systems. In general, the higher the score function value, the better the comprehensive conditions of the corresponding Distributed Control System. According to the Cq-ROFHSEWA operator or the Cq-ROFHSEWG operator, the score function value ranking is as follows:

Y^(5)^ > Y^(4)^ > Y^(2)^ > Y^(1)^ > Y^(3)^ or Y^(5)^ > Y^(4)^ > Y^(2)^ > Y^(3)^ > Y^(1)^.

As can be seen, the Distributed Control System with the best comprehensive performance is Y^(5)^. Notice that the both operators provide different results. The Cq-ROFHSEWA operator shows that Y^(1)^ is the fourth choice and Y^(3)^ is the last option, but the Cq-ROFHSEWG operator shows that Y^(3)^ is the fourth choice and Y^(1)^ is the last choice option.

## 5. Analysis and Discussion of Comparative Studies

As we discuss in this section, the proposed method is evaluated in terms of its effectiveness, simplicity, operability, and benefits. Additionally, we present a brief comparison between the proposed method and some prior art.

### 5.1. The Superiority of the Proposed Method

Zadeh’s FS [1] provides inaccurate and imprecise membership information but does not provide information regarding the NMem of alternatives under the parameters considered. Currently, FS uses only Mem to resolve difficulties, whereas our proposed technique makes use of the inherent ambiguity in both Mem and NMem. Atanassov [2] uses Mem and NMem to address uncertainty in their IFS. This theory cannot, however, be applied in situations where the sum of Mem and NMem exceeds 1. When compared with the IFS, the PFS of Yager [10,12] is able to accommodate a greater degree of uncertainty. These theories do not take into account the parameters of the alternatives. The concept of FSS was developed in order to address the problem of parameterizing uncertain objects, according to Maki et al. [23]. A consideration of the Mem of the attributes is necessary in order to deal with uncertainty. Nevertheless, the proposed FSS does not provide any information regarding the object’s NMem. Maji et al. [24] have proposed using IFSS to overcome these drawbacks. IFSS is unable to handle cases where Mem and NMem exceed one, but our proposal overcomes these obstacles and provides more operational benefits. Cq-ROFHSS is a special case of hybrid structures such as FS and IFS when certain conditions are met. Any of the studies mentioned above provide no information regarding the sub-properties. Accordingly, the above-mentioned theories cannot explain the case in which attributes have corresponding subproperties. In addition to providing additional operational results to the MADM method, our proposed approach addresses these complex issues as well.

The proposed concept allows for a more accurate handling of uncertain objects. The MADM process can be quite effective when entity information is accurately and empirically represented (see Table 6). On the basis of the results of this study and comparison, it has been determined that the proposed method produces more accurate results than other methods. In addition, MADM can incorporate a significant amount of information to address anxiety in the data, as opposed to other methods. As a result of its effectiveness, flexibility, simplicity, and superior performance, our hybrid structure performs better than other hybrid structures for fuzzy sets.

### 5.2. Comparative Analysis

#### 5.2.1. Influence of Parameter Values on Ranking Results

This section discusses the effects of parameter *q* on the ranking results of the alternatives. First, we adjusted the parameter *q* using Equations (11) and (12) in order to produce a more accurate representation of the warning system information. In order to investigate the effect of different values of *q* = 2, 3, 4, 5, 8, 10, 15, and 20 on the final ranking results, the analytical calculations were repeated as shown in Table 7 and Table 8.

Following that, we examined the effect of parameters on the results of the alternatives. In Table 7 and Table 8, the parameters have an impact on the best ranking results, and the value of the score function gradually changes with an increase in parameter *q*. As an example, the score function of the Cq-ROFHSEWA aggregation operator gradually increases with increases in parameter *q*, while the score function of the Cq-ROFHSEWG aggregation operator gradually decreases with increases in parameter *q*. However, the best ranking results remain unchanged.

**Figure 5:**
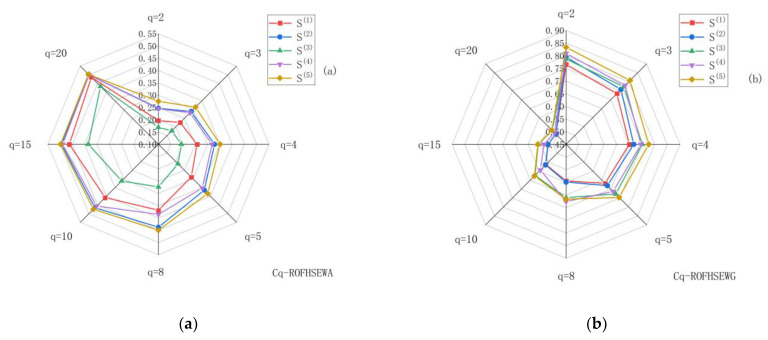
Geometric interpretation of the proposed work ((**a**): The effect of the parameter *q*, from the Cq-ROFHSEWA operator. (**b**): The effect of the parameter *q*, from the Cq-ROFHSEWG operator).

Figure 5 presents a geometrical interpretation of the proposed work described in Table 7 and Table 8.

According to Figure 5, the decision maker has the option of selecting different aggregation operators and changing the results of each scenario by modifying the adjustment parameter *q*. It should be noted, however, that the best ranking results remain unchanged. Therefore, it shows that the complex q-rung orthopair fuzzy hypersoft information integration operator proposed in this paper is feasible and internally consistent.

#### 5.2.2. Comparison with Existing Methods

According to [30], Zulqarnain et al. propose intuitionistic fuzzy hypersoft sets (IFHSS), as well as new aggregation operators, an intuitionistic fuzzy hypersoft weighted average (IFHWA) operator, and an intuitionistic fuzzy hypersoft weighted geometric (IFHWG) operator, and discuss some of their properties. On the basis of the proposed operators, a decision method is developed to solve the MADM problem. However, this method cannot be used in situations where the sum of the squares of (interval) Mem and (interval) NMem exceeds 1. Additionally, they [25] proposed the concept of the Pythagorean fuzzy soft set (PFSS) and derived from it the Pythagorean fuzzy soft weighted average (PFSWA) operator and Pythagorean fuzzy soft weighted geometric (PCFWG) operator. As a result of these operators, Pythagorean fuzzy soft information can be used to solve decision problems. This method cannot be applied if the information is the complex Mem and complex NMem. Liu et al. [19] propose the complex q-rung orthopair fuzzy weighted average operator (Cq-ROFWA) and complex q-rung orthopair fuzzy weighted geometric operator (Cq-ROFWG) to solve the MCDM problem. However, a major common limitation of this theory is that they are not suitable for parametric descriptions. 

In this paper, we propose a method that is capable of handling this situation easily. Through the parameter *q*, the method proposed in this paper is more flexible than the methods proposed in [30] and [25]. In order to avoid information distortion, the scope of the decision process can be broadened by increasing the value of the parameter *q*. As a result, the method proposed in this paper is more suitable, since it gives the decision maker more space in the decision-making process. Furthermore, the method presented in this paper has the advantage of being capable of handling parametric descriptions, which is an advantage over [19].

Complex q-rung orthopair fuzzy hypersoft sets (Cq-ROFHSSs) provide an effective description of complex fuzzy information in the real world. Since Cq-ROFHSSs contain the parameter *q* and can adjust the range of complex fuzzy information expressed, the Einstein aggregation operator is a useful tool for generating rules based on complex q-rung orthopair fuzzy hypersoft numbers (Cq-ROFHSNs). The proposed theory resolves the parametric ambiguity of two-dimensional fuzzy data, which makes it superior to the Cq-ROFS model from the beginning. We have conducted a comparative analysis of the seminal approach to illustrate its usefulness and superiority. The same examples are solved using some existing methods, and their results are evaluated.

As part of this section, we make a number of quantitative comparisons. The same examples are solved using existing methods, and their final ranking results are compared. Using our pioneering method, we compare it to the methods proposed by Zulqarnain [30] for the weighted intuitionistic fuzzy hypersoft aggregation operators (IFHWA, IFHWG), and Pythagorean fuzzy soft weighted averaging operators (PFSWA, PFSWG) [25], as well as Liu’s complex q-rung orthopair fuzzy weighted averaging operators (Cq-ROFWA, Cq-ROFWAG) [19]. A practical multi-attribute decision example is used in this paper in order to demonstrate the superiority of the proposed method.

**Example** **5.** *Consider a very simple decision-making problem: ranking the performance of distributed control systems. This example differs from Example 4 in that the experts provide their preferences as intuitionistic fuzzy numbers. In the previous section, we discussed the information related to this example. Table 9, Table 10 and Table 11 provide a description of intuitionistic fuzzy information.*

This algorithm consists of the following steps:

**Step 1.** Each entity is represented as an intuitionistic fuzzy number in this example. After clarifying that $e^{0}=1$, we constructed a matrix in which each entity is represented by a complex number.

**Step 2.** No need for normalization because all parameters are of the same type. 

**Step 3.** Based on the assumption that *q* = 1, determine the preference values of attributes by using the Cq-ROFHSEWA or Cq-ROFHSEWG operators, and determine the summary matrix as shown in Table 10 and Table 11.

**Step 4.** Using the Cq-ROFHSEWA operator, compute the corresponding score function values for each distributed control system:

Ş^(1)^ = 0.3445, Ş^(2)^ = 0.3585, Ş^(3)^ = 0.3531, Ş^(4)^ = 0.3629, Ş^(5)^ = 0.3430,

From the Cq-ROFHSEWG operator, calculate the score function values between each alternative:

Ş^(1)^ = 0.6558, Ş^(2)^ = 0.6701, Ş^(3)^ = 0.6663, Ş^(4)^ = 0.6750, Ş^(5)^ = 0.6581.

**Step 5.** According to the score function value, rank the advantages and disadvantages of the five Distributed Control Systems. Generally speaking, the higher the score function value, the better the comprehensive conditions of the Distributed Control System. From either the Cq-ROFHSEWA operator or the Cq-ROFHSEWG operator, the following score function value ranking is provided:

Y^(4)^ > Y^(2)^ > Y^(3)^ > Y^(1)^ > Y^(5)^ or Y^(4)^ > Y^(2)^ > Y^(3)^ > Y^(5)^ > Y^(1)^.

As can be seen, the Distributed Control System with the best comprehensive performance is Y^(4)^. It should be noted that the results provided by the two operators are different. According to the Cq-ROFHSEWA operator, it shows that Y^(1)^ is the fourth option and Y^(5)^ is the last option, whereas, according to the Cq-ROFHSEWG operator, it shows that Y^(5)^ is the fourth option and Y^(1)^ is the last option.

The following section compares the proposed method with the results of the existing operators IFHWA, IFHWG [30], PFSWA, PFSWG [25], Cq-ROFWA, and Cq-ROFWG [19] for Example 5. In the following section, we will demonstrate that the proposed Cq-ROFHSEWA and Cq-ROFHSEWG operators generate more fruitful and more general results than the existing operators.

**Figure 6:**
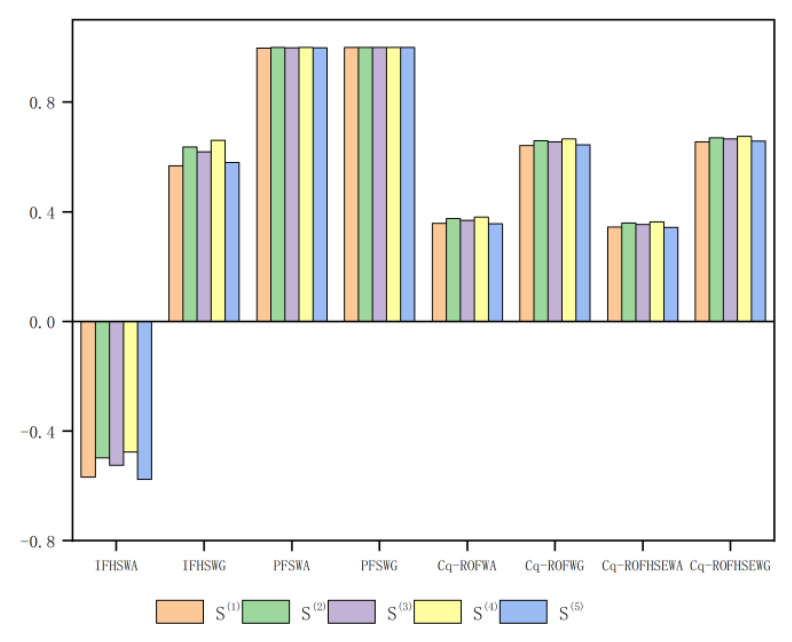
The geometrical interpretation of the proposed work.

In Table 12, the ranking results obtained by the existing methods and the proposed methods are compared according to Example 5. Based on the comparison in Table 12, Figure 6 provides a geometric explanation.

The geometrical interpretation of the proposed work described in Table 12 are available in Figure 6.

It is evident from Table 12 and Figure 6 that the same order of these methods gives different results, but the best choice is Y^(4)^. It will be found that IFHSWA [30], PFSWG [25], Cq-ROFWA [19], and Cq-ROFHSEWA all yield the same ranking of results, while IFHSWG [30], PFSWA [25], Cq-ROFWG [19], and Cq-ROFHSEWG also yield the same ranking of results.

Despite this, IFHSS [30] cannot adequately describe the problem, whereas Cq-ROFHSSs are more capable of handling uncertain and unpredictable information in realistic decision-making situations. In order to further discuss our proposed method, we will examine its superiority and flexibility by using different values of parameter q. If we consider the Cq-ROFHSSs type, IFHSS [30] and CPFSS [19] cannot adequately describe it. It is not possible to meet the conditions of IFHSS and CPFSS in an efficient manner. 

To demonstrate the effectiveness of the proposed method, we change the imaginary part of all sets in Example 4 to 0 (IM = 0), resulting in a simplified Complex q-rung orthopair Fuzzy Hypersoft Set. This allows comparing Zulqarnain’s two weighted intuitionistic fuzzy hypersoft leveling operators (IFHWA, IFHWG) [30] and Pythagorean fuzzy soft weighted averaging operators (PFSWA, PFSWG) [25], as well as Liu’s complex q-rung orthopair fuzzy weighted averaging operators (Cq-ROFWA, Cq-ROFWAG) [19]. A summary of the results can be found in Table 13. 

As shown in Table 13, the same order of these methods yields three different results, but Y^(5)^ is the most appropriate choice. The operators of IFHSWA, IFHSWG [30], and PFSWG [25] indicate that Y^(4)^ is the second option and Y^(1)^ is the last option, whereas the operators of PFSWA [25], Cq-ROFWG [19], and Cq-ROFHSEWG indicate that Y^(3)^ is the second choice and Y^(1)^ is the last choice. In Cq-ROFWA [19], Cq-ROFHSEWA shows that Y^(4)^ is the second choice, while Y^(3)^ is the last option.

**Figure 7:**
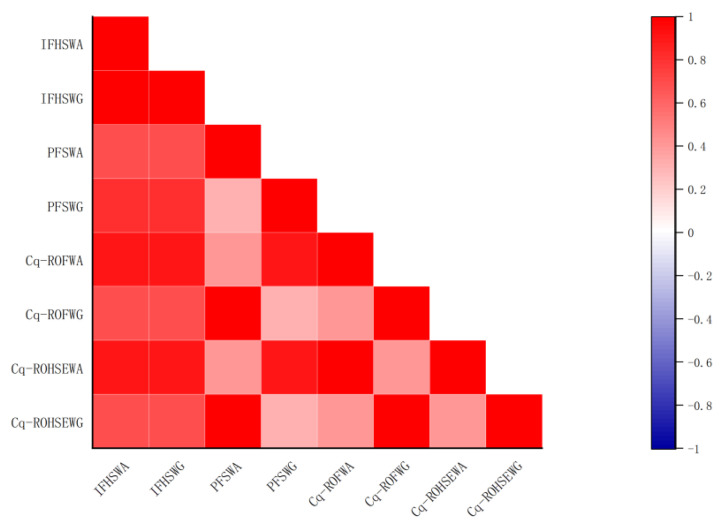
Spearman correlation plot.

Following that, Table 14 presents the different ranking results derived from different methods in the future. Based on the ranking in Table 14, a Spearman correlation analysis is conducted in order to obtain Figure 7.

Figure 7 illustrates the Spearman correlations (see Table 14) based on different methods and ranking orders [48]. The proposed framework is highly compatible with other state-of-the-art approaches based on the analysis of the Spearman rank coefficients shown in Figure 7. Despite this, the proposed set of ranking values is more reasonable as it also incorporates complex fuzzy information and sub-attributes.

In comparison with existing methods, the proposed method has the following advantages:

(i) The proposed approach is highly complementary to current state-of-the-art techniques. It is clear from Figure 7 that this conclusion can be drawn.
(ii) It is assumed that the sum of the q powers of the Mem degree and the NMem degree is constrained to the unit circle within the complex plane. IFHSSs and PFSSs cannot handle complex information provided by decision makers. Cq-ROFHSSs can provide a solution to this problem.
(iii) The proposed approach is more general than the Cq-ROFS approaches. A wide variety of practical applications are possible by subdividing attributes into subattributes. The proposed theory addresses the parametric ambiguity of two-dimensional fuzzy data, making it superior to the Cq-ROFS models since its inception.
(iv) One of the principal advantages of the proposed MADM approach is its ability to examine much more relevant data to approach the alternative with less data loss. As well, employing the different values of *q* will provide a relief to the decision expert in choosing the best alternative(s) with the field relaxation. Due to this, it can be interpreted that by utilizing different choices for the dataset, the presented generalized aggregation model assists decision experts in making the best decision.
(v) An example from an early warning system for public opinion illustrates the practicality and effectiveness of the framework.

According to the comparison above, the methods proposed in this paper, such as Cq-ROFHSEWA and Cq-ROFHSEWG, are more general than IFHSS, PFSS, and Cq-ROFS. In this paper, a method is proposed that is more suitable for solving multi-attribute decision problems. Despite the fact that this proposal has several advantages, it also has some disadvantages. Since this algorithm utilizes information from Cq-ROFHSSs, it has a higher complexity than its counterpart.

## 6. Conclusions

This article establishes the basis for a multi-skill mixed model known as the Cq-ROFHSS. This tool is capable of capturing all types of errors in human cognition. It provides a mathematical framework for representing two-dimensional inaccurate information in a flexible and competent manner. Therefore, it extends several contemporary models by incorporating the expertise of a Complex q-rung orthopair Fuzzy Set and the remarkable parameters of HSSs. As a result of this theory, CIFHSSs and CPFHSSs are powerfully generalized. The result of this study is a formal definition of Cq-ROFHSS and its basic set theory operation. Additionally, we propose some algebraic operations that can be carried out by the Einstein operation in addition to Cq-ROFHSS. The rationality of our model has been demonstrated, and its relationship with existing theories has been briefly discussed.

Moreover, this research contributes to the development of two MADM algorithms in the Cq-ROFHSS environment, which are used to identify the most optimal alternatives based on their intrinsic characteristics. As a result of applying these methods in practice, we have been able to determine which DCS is the most suitable in terms of performance. An analysis of contemporary MADM technology was conducted in order to demonstrate that our strategy is a reasonable extension of the technology. Our comparative study was accompanied by an explanatory bar chart and Spearman analysis that illustrated the compatibility and accuracy of the final results. Furthermore, we are committed to reducing the number of calculations of these MADM technologies through some computer programming and by developing a graphical representation of the proposed model in order to better explain this skilled concept. To demonstrate its superiority to existing decision theories, we examine the dynamic characteristics of the proposed model.

In spite of the fact that the developed model has some advantages over contemporary methods, it is not without limitations. Consequently, its structure cannot convey the abstinence and rejection aspects of inaccurate human expressions. Furthermore, since our strategies for solving the MADM problem require tedious and difficult calculations, they may be computationally intensive. The development of more advanced MADM strategies will be the focus of our future research, such as the Cq-ROFHSS-PROMETHE method, the Cq-ROFHSS-VIKOR method, the Cq-ROFHSS-AHP method, and the Cq-ROFHSS-ELECTRE method. Our intention is to explore the potential application scope of the Cq-ROFHSS model in a variety of environments.

## Figures and Tables

**Table 1:** Cq-ROFHSNs decision matrix for $u_{1}$.

	sub-attributes ${\dot{f}}_{i}$				
	${\dot{f}}_{1}$	${\dot{f}}_{2}$	${\dot{f}}_{3}$	${\dot{f}}_{4}$	${\dot{f}}_{5}$
Y^(1)^	(0.36e^i2π0.74^,0.79e^i2π0.69^)	(0.56e^i2π0.58^,0.95e^i2π0.8^)	(0.56e^i2π0.36^,0.75e^i2π0.83^)	(0.71e^i2π0.47^,0.66e^i2π0.57^)	(0.92e^i2π0.93^,0.35e^i2π0.47^)
Y^(2)^	(0.38e^i2π0.91^,0.51e^i2π0.8^)	(0.22e^i2π0.95^,0.88e^i2π0.7^)	(0.41e^i2π0.92^,0.76e^i2π0.29^)	(0.37e^i2π0.48^,0.23e^i2π0.91^)	(0.64e^i2π0.74^,0.25e^i2π0.36^)
Y^(3)^	(0.59e^i2π0.77^,0.47e^i2π0.55^)	(0.93e^i2π0.42^,0.83e^i2π0.46^)	(0.51e^i2π0.75^,0.44e^i2π0.93^)	(0.87e^i2π0.83^,0.45e^i2π0.86^)	(0.8e^i2π0.81^,0.54e^i2π0.87^)
Y^(4)^	(0.47e^i2π0.84^,0.4e^i2π0.9^)	(0.3e^i2π0.7^,0.42e^i2π0.45^)	(0.76e^i2π0.48^,0.24e^i2π0.79^)	(0.68e^i2π0.62^,0.84e^i2π0.64^)	(0.33e^i2π0.93^,0.36e^i2π0.37^)
Y^(5)^	(0.52e^i2π0.92^,0.32e^i2π0.79^)	(0.36e^i2π0.31^,0.78e^i2π0.64^)	(0.46e^i2π0.63^,0.31e^i2π0.79^)	(0.6e^i2π0.43^,0.44e^i2π0.41^)	(0.67e^i2π0.8^,0.86e^i2π0.32^)
	sub-attributes ${\dot{f}}_{i}$				
	${\dot{f}}_{6}$	${\dot{f}}_{7}$	${\dot{f}}_{8}$	${\dot{f}}_{9}$	
Y^(1)^	(0.66e^i2π0.69^,0.28e^i2π0.3^)	(0.81e^i2π0.65^,0.93e^i2π0.69^)	(0.28e^i2π0.93^,0.33e^i2π0.3^)	(0.61e^i2π0.63^,0.53e^i2π0.39^)	
Y^(2)^	(0.94e^i2π0.68^,0.43e^i2π0.57^)	(0.63e^i2π0.6^,0.72e^i2π0.5^)	(0.22e^i2π0.6^,0.32e^i2π0.74^)	(0.43e^i2π0.8^,0.47e^i2π0.68^)	
Y^(3)^	(0.73e^i2π0.64^,0.55e^i2π0.4^)	(0.86e^i2π0.85^,0.7e^i2π0.95^)	(0.6e^i2π0.93^,0.93e^i2π0.54^)	(0.46e^i2π0.68^,0.67e^i2π0.8^)	
Y^(4)^	(0.63e^i2π0.62^,0.81e^i2π0.65^)	(0.88e^i2π0.85^,0.53e^i2π0.92^)	(0.36e^i2π0.57^,0.32e^i2π0.68^)	(0.65e^i2π0.29^,0.37e^i2π0.75^)	
Y^(5)^	(0.38e^i2π0.92^,0.28e^i2π0.77^)	(0.87e^i2π0.37^,0.87e^i2π0.75^)	(0.94e^i2π0.61^,0.7e^i2π0.23^)	(0.76e^i2π0.79^,0.52e^i2π0.68^)	

**Table 2:** Cq-ROFHSNs decision matrix for $u_{2}$.

	sub-attributes ${\dot{f}}_{i}$				
	${\dot{f}}_{1}$	${\dot{f}}_{2}$	${\dot{f}}_{3}$	${\dot{f}}_{4}$	${\dot{f}}_{5}$
Y^(1)^	(0.38e^i2π0.27^,0.48e^i2π0.87^)	(0.35e^i2π0.46^,0.45e^i2π0.53^)	(0.21e^i2π0.5^,0.74e^i2π0.73^)	(0.52e^i2π0.86^,0.77e^i2π0.91^)	(0.37e^i2π0.34^,0.92e^i2π0.29^)
Y^(2)^	(0.58e^i2π0.28^,0.86e^i2π0.23^)	(0.53e^i2π0.84^,0.57e^i2π0.42^)	(0.5e^i2π0.24^,0.21e^i2π0.67^)	(0.39e^i2π0.23^,0.61e^i2π0.56^)	(0.63e^i2π0.71^,0.26e^i2π0.61^)
Y^(3)^	(0.41e^i2π0.95^,0.68e^i2π0.54^)	(0.54e^i2π0.43^,0.55e^i2π0.63^)	(0.85e^i2π0.71^,0.78e^i2π0.83^)	(0.61e^i2π0.65^,0.69e^i2π0.89^)	(0.83e^i2π0.54^,0.83e^i2π0.88^)
Y^(4)^	(0.26e^i2π0.93^,0.77e^i2π0.42^)	(0.91e^i2π0.43^,0.94e^i2π0.37^)	(0.59e^i2π0.62^,0.88e^i2π0.34^)	(0.25e^i2π0.38^,0.21e^i2π0.25^)	(0.43e^i2π0.21^,0.56e^i2π0.54^)
Y^(5)^	(0.35e^i2π0.68^,0.86e^i2π0.31^)	(0.53e^i2π0.73^,0.46e^i2π0.3^)	(0.2e^i2π0.45^,0.3e^i2π0.59^)	(0.29e^i2π0.26^,0.68e^i2π0.46^)	(0.81e^i2π0.84^,0.22e^i2π0.85^)
	sub-attributes ${\dot{f}}_{i}$				
	${\dot{f}}_{6}$	${\dot{f}}_{7}$	${\dot{f}}_{8}$	${\dot{f}}_{9}$	
Y^(1)^	(0.42e^i2π0.91^,0.22e^i2π0.4^)	(0.22e^i2π0.44^,0.23e^i2π0.72^)	(0.36e^i2π0.69^,0.88e^i2π0.31^)	(0.77e^i2π0.53^,0.33e^i2π0.83^)	
Y^(2)^	(0.55e^i2π0.3^,0.86e^i2π0.21^)	(0.95e^i2π0.24^,0.44e^i2π0.32^)	(0.21e^i2π0.78^,0.24e^i2π0.41^)	(0.47e^i2π0.94^,0.53e^i2π0.29^)	
Y^(3)^	(0.69e^i2π0.71^,0.89e^i2π0.87^)	(0.78e^i2π0.76^,0.82e^i2π0.86^)	(0.49e^i2π0.63^,0.85e^i2π0.58^)	(0.89e^i2π0.71^,0.69e^i2π0.72^)	
Y^(4)^	(0.53e^i2π0.29^,0.55e^i2π0.3^)	(0.32e^i2π0.86^,0.86e^i2π0.51^)	(0.68e^i2π0.87^,0.24e^i2π0.45^)	(0.64e^i2π0.63^,0.24e^i2π0.31^)	
Y^(5)^	(0.69e^i2π0.81^,0.45e^i2π0.38^)	(0.65e^i2π0.36^,0.27e^i2π0.53^)	(0.91e^i2π0.88^,0.25e^i2π0.23^)	(0.67e^i2π0.63^,0.25e^i2π0.15^)	

**Table 3:** Cq-ROFHSNs decision matrix for $u_{3}$.

	sub-attributes ${\dot{f}}_{i}$				
	${\dot{f}}_{1}$	${\dot{f}}_{2}$	${\dot{f}}_{3}$	${\dot{f}}_{4}$	${\dot{f}}_{5}$
Y^(1)^	(0.93e^i2π0.86^,0.2e^i2π0.42^)	(0.61e^i2π0.5^,0.51e^i2π0.56^)	(0.85e^i2π0.75^,0.78e^i2π0.48^)	(0.39e^i2π0.3^,0.7e^i2π0.25^)	(0.67e^i2π0.47^,0.86e^i2π0.59^)
Y^(2)^	(0.76e^i2π0.71^,0.21e^i2π0.43^)	(0.65e^i2π0.49^,0.88e^i2π0.63^)	(0.79e^i2π0.84^,0.2e^i2π0.43^)	(0.62e^i2π0.2^,0.61e^i2π0.5^)	(0.91e^i2π0.27^,0.58e^i2π0.28^)
Y^(3)^	(0.6e^i2π0.72^,0.67e^i2π0.86^)	(0.4e^i2π0.62^,0.75e^i2π0.6^)	(0.66e^i2π0.54^,0.51e^i2π0.5^5)	(0.5e^i2π0.67^,0.88e^i2π0.79^)	(0.88e^i2π0.45^,0.57e^i2π0.75^)
Y^(4)^	(0.24e^i2π0.66^,0.5e^i2π0.66^)	(0.27e^i2π0.23^,0.82e^i2π0.52^)	(0.81e^i2π0.36^,0.51e^i2π0.85^)	(0.62e^i2π0.33^,0.62e^i2π0.92^)	(0.89e^i2π0.56^,0.34e^i2π0.33^)
Y^(5)^	(0.6e^i2π0.87^,0.87e^i2π0.95^)	(0.44e^i2π0.45^,0.37e^i2π0.31^)	(0.74e^i2π0.63^,0.42e^i2π0.74^)	(0.82e^i2π0.94^,0.93e^i2π0.23^)	(0.83e^i2π0.39^,0.75e^i2π0.57^)
	sub-attributes ${\dot{f}}_{i}$				
	${\dot{f}}_{6}$	${\dot{f}}_{7}$	${\dot{f}}_{8}$	${\dot{f}}_{9}$	
Y^(1)^	(0.71e^i2π0.35^,0.81e^i2π0.9^)	(0.87e^i2π0.65^,0.71e^i2π0.8^3)	(0.22e^i2π0.74^,0.57e^i2π0.95^)	(0.25e^i2π0.32^,0.72e^i2π0.83^)	
Y^(2)^	(0.27e^i2π0.9^,0.84e^i2π0.38^)	(0.76e^i2π0.3^,0.68e^i2π0.56^)	(0.31e^i2π0.6^,0.37e^i2π0.25^)	(0.27e^i2π0.53^,0.73e^i2π0.34^)	
Y^(3)^	(0.57e^i2π0.4^,0.73e^i2π0.92^)	(0.53e^i2π0.51^,0.63e^i2π0.95^)	(0.53e^i2π0.43^,0.61e^i2π0.61^)	(0.49e^i2π0.49^,0.64e^i2π0.65^)	
Y^(4)^	(0.54e^i2π0.37^,0.51e^i2π0.64^)	(0.69e^i2π0.64^,0.5e^i2π0.28^)	(0.95e^i2π0.79^,0.27e^i2π0.69^)	(0.69e^i2π0.94^,0.72e^i2π0.23^)	
Y^(5)^	(0.26e^i2π0.86^,0.25e^i2π0.54^)	(0.3e^i2π0.51^,0.59e^i2π0.86^)	(0.83e^i2π0.87^,0.44e^i2π0.27^)	(0.89e^i2π0.7^,0.62e^i2π0.63^)	

**Table 4:** Cq-ROFHSEWA operator’s overall assessment of each alternative.

Alternatives	Cq-ROFHSEWA
Y^(1)^	(0.1978e^i2π0.2478^,0.8453e^i2π0.8562^)
Y^(2)^	(0.1956e^i2π0.265^8,0.8059e^i2π0.7855^)
Y^(3)^	(0.2626e^i2π0.2666^,0.8974e^i2π0.9028^)
Y^(4)^	(0.246e^i2π0.2606^,0.8047e^i2π0.8104^)
Y^(5)^	(0.2748e^i2π0.2964^,0.7938e^i2π0.7869^)

**Table 5:** Cq-ROFHSEWG operator’s overall assessment of each alternative.

Alternatives	Cq-ROFHSEWG
Y^(1)^	(0.8011e^i2π0.8484^,0.2536e^i2π0.2735^)
Y^(2)^	(0.8e^i2π0.8413^,0.2058e^i2π0.1677^)
Y^(3)^	(0.8705e^i2π0.8745^,0.2963e^i2π0.3163^)
Y^(4)^	(0.8449e^i2π0.8486^,0.2086e^i2π0.2009^)
Y^(5)^	(0.858e^i2π0.8826^,0.1893e^i2π0.1867^)

**Table 6:** Comparison of Cq-ROFHSSs with some existing theories.

SN	References	Set	Truth Information	Falsity	Parameterization	Attributes	Sub-Attributes	Limitations
1	Zadeh [1]	FS	√	✘	✘	√	✘	Lack of NMem
2	Atanassov [2]	IFS	√	√	✘	√	✘	Lack of complex fuzzy values
3	Yager [10]	PFS	√	√	✘	√	✘	Lack of complex fuzzy values
4	Yager [12]	q-ROFS	√	√	✘	√	✘	Lack of complex fuzzy values
5	Maji et al. [23]	FSS	√	✘	√	√	✘	Cannot deal with NMem of the parameters
6	Maji et al. [24]	IFSS	√	√	√	√	✘	Lack of complex fuzzy values
7	Proposed Approach	Cq-ROFHSS	√	√	√	√	√	Long and heavy calculations in grades diagnosis

**Table 7:** Effect of the parameter *q* from the Cq-ROFHSWA operator.

Value of *q*	The Result of the Methods	Rankings of the Methods
*q* = 2	Ş^(1)^ = 0.1965, Ş^(2)^ = 0.2465, Ş^(3)^ = 0.1689, Ş^(4)^ = 0.2446, Ş^(5)^ = 0.2749	Y^(5)^ > Y^(4)^ > Y^(2)^ > Y^(1)^ > Y^(3)^
*q* = 3	Ş^(1)^ = 0.225, Ş^(2)^ = 0.2881, Ş^(3)^ = 0.1773, Ş^(4)^ = 0.2812, Ş^(5)^ = 0.3137	Y^(5)^ > Y^(2)^ > Y^(4)^ > Y^(1)^ > Y^(3)^
*q* = 4	Ş^(1)^ = 0.2573, Ş^(2)^ = 0.3285, Ş^(3)^ = 0.1934, Ş^(4)^ = 0.3185, Ş^(5)^ = 0.3511	Y^(5)^ > Y^(2)^ > Y^(4)^ > Y^(1)^ > Y^(3)^
*q* = 5	Ş^(1)^ = 0.289, Ş^(2)^ = 0.3641, Ş^(3)^ = 0.2127, Ş^(4)^ = 0.3524, Ş^(5)^ = 0.3838	Y^(5)^ > Y^(2)^ > Y^(4)^ > Y^(1)^ > Y^(3)^
*q* = 8	Ş^(1)^ = 0.3686, Ş^(2)^ = 0.4379, Ş^(3)^ = 0.2739, Ş^(4)^ = 0.3856, Ş^(5)^ = 0.4501	Y^(5)^ > Y^(2)^ > Y^(4)^ > Y^(1)^ > Y^(3)^
*q* = 10	Ş^(1)^ = 0.4068, Ş^(2)^ = 0.4655, Ş^(3)^ = 0.3115, Ş^(4)^ = 0.4558, Ş^(5)^ = 0.474	Y^(5)^ > Y^(2)^ > Y^(4)^ > Y^(1)^ > Y^(3)^
*q* = 15	Ş^(1)^ = 0.4625, Ş^(2)^ = 0.4947, Ş^(3)^ = 0.3854, Ş^(4)^ = 0.4897, Ş^(5)^ = 0.498	Y^(5)^ > Y^(2)^ > Y^(4)^ > Y^(1)^ > Y^(3)^
*q* = 20	Ş^(1)^ = 0.4857, Ş^(2)^ = 0.5014, Ş^(3)^ = 0.4330, Ş^(4)^ = 0.4990, Ş^(5)^ = 0.5025	Y^(5)^ > Y^(2)^ > Y^(4)^ > Y^(1)^ > Y^(3)^

**Table 8:** Effect of the parameter *q* from the Cq-ROFHSWG operator.

Value of *q*	The Result of the Methods	Rankings of the Methods
*q* = 2	Ş^(1)^ = 0.7654, Ş^(2)^ = 0.7946, Ş^(3)^ = 0.7772, Ş^(4)^ = 0.809, Ş^(5)^ = 0.8334	Y^(5)^ > Y^(4)^ > Y^(2)^ > Y^(3)^ > Y^(1)^
*q* = 3	Ş^(1)^ = 0.7336, Ş^(2)^ = 0.7565, Ş^(3)^ = 0.772, Ş^(4)^ = 0.7792, Ş^(5)^ = 0.807	Y^(5)^ > Y^(4)^ > Y^(3)^ > Y^(2)^ > Y^(1)^
*q* = 4	Ş^(1)^ = 0.6997, Ş^(2)^ = 0.7171, Ş^(3)^ = 0.7507, Ş^(4)^ = 0.7459, Ş^(5)^ = 0.7765	Y^(5)^ > Y^(3)^ > Y^(4)^ > Y^(2)^ > Y^(1)^
*q* = 5	Ş^(1)^ = 0.6678, Ş^(2)^ = 0.6808, Ş^(3)^ = 0.7274, Ş^(4)^ = 0.7132, Ş^(5)^ = 0.746	Y^(5)^ > Y^(3)^ > Y^(4)^ > Y^(2)^ > Y^(1)^
*q* = 8	Ş^(1)^ = 0.5944, Ş^(2)^ = 0.599, Ş^(3)^ = 0.6603, Ş^(4)^ = 0.6759, Ş^(5)^ = 0.6669	Y^(5)^ > Y^(3)^ > Y^(4)^ > Y^(2)^ > Y^(1)^
*q* = 10	Ş^(1)^ = 0.5626, Ş^(2)^ = 0.5647, Ş^(3)^ = 0.6233, Ş^(4)^ = 0.5933, Ş^(5)^ = 0.6266	Y^(5)^ > Y^(3)^ > Y^(4)^ > Y^(2)^ > Y^(1)^
*q* = 15	Ş^(1)^ = 0.5213, Ş^(2)^ = 0.5217, Ş^(3)^ = 0.5603, Ş^(4)^ = 0.5374, Ş^(5)^ = 0.5614	Y^(5)^ > Y^(3)^ > Y^(4)^ > Y^(2)^ > Y^(1)^
*q* = 20	Ş^(1)^ = 0.5068, Ş^(2)^ = 0.5072, Ş^(3)^ = 0.5280, Ş^(4)^ = 0.5144, Ş^(5)^ = 0.5291	Y^(5)^ > Y^(3)^ > Y^(4)^ > Y^(2)^ > Y^(1)^

**Table 9:** Decision matrix Cq-ROFHSSs for $u_{i}$, whose entities are derived from intuitionistic fuzzy numbers.

Information given by expert $u_{1}$	sub-attributes ${\dot{f}}_{i}$								
	${\dot{f}}_{1}$	${\dot{f}}_{2}$	${\dot{f}}_{3}$	${\dot{f}}_{4}$	${\dot{f}}_{5}$	${\dot{f}}_{6}$	${\dot{f}}_{7}$	${\dot{f}}_{8}$	${\dot{f}}_{9}$
Y^(1)^	(0.52, 0.33)	(0.37, 0.37)	(0.24, 0.28)	(0.4, 0.43)	(0.52, 0.34)	(0.51, 0.42)	(0.38, 0.43)	(0.5, 0.2)	(0.44, 0.5)
Y^(2)^	(0.31, 0.38)	(0.54, 0.45)	(0.27, 0.31)	(0.44, 0.33)	(0.46, 0.31)	(0.65, 0.21)	(0.52, 0.42)	(0.33, 0.44)	(0.63, 0.31)
Y^(3)^	(0.5, 0.26)	(0.49, 0.33)	(0.62, 0.33)	(0.36, 0.62)	(0.72, 0.22)	(0.43, 0.48)	(0.37, 0.47)	(0.55, 0.31)	(0.35, 0.41)
Y^(4)^	(0.33, 0.31)	(0.48, 0.35)	(0.31, 0.63)	(0.71, 0.21)	(0.53, 0.4)	(0.43, 0.53)	(0.38, 0.32)	(0.66, 0.28)	(0.55, 0.33)
Y^(5)^	(0.54, 0.28)	(0.34, 0.36)	(0.43, 0.51)	(0.32, 0.56)	(0.22, 0.59)	(0.5, 0.42)	(0.49, 0.36)	(0.38, 0.43)	(0.46, 0.38)
Information given by expert $u_{2}$	sub-attributes ${\dot{f}}_{i}$								
	${\dot{f}}_{1}$	${\dot{f}}_{2}$	${\dot{f}}_{3}$	${\dot{f}}_{4}$	${\dot{f}}_{5}$	${\dot{f}}_{6}$	${\dot{f}}_{7}$	${\dot{f}}_{8}$	${\dot{f}}_{9}$
Y^(1)^	(0.51, 0.48)	(0.21, 0.49)	(0.54, 0.32)	(0.53, 0.24)	(0.33, 0.24)	(0.32, 0.26)	(0.27, 0.24)	(0.34, 0.32)	(0.45, 0.43)
Y^(2)^	(0.54, 0.42)	(0.41, 0.27)	(0.3, 0.42)	(0.66, 0.28)	(0.71, 0.22)	(0.35, 0.55)	(0.72, 0.2)	(0.53, 0.35)	(0.52, 0.35)
Y^(3)^	(0.39, 0.53)	(0.42, 0.41)	(0.67, 0.2)	(0.39, 0.41)	(0.29, 0.49)	(0.46, 0.35)	(0.43, 0.38)	(0.69, 0.25)	(0.41, 0.51)
Y^(4)^	(0.59, 0.26)	(0.21, 0.48)	(0.54, 0.21)	(0.42, 0.55)	(0.65, 0.31)	(0.47, 0.34)	(0.57, 0.34)	(0.5, 0.48)	(0.63, 0.27)
Y^(5)^	(0.4, 0.49)	(0.29, 0.4)	(0.44, 0.47)	(0.55, 0.27)	(0.7, 0.21)	(0.4, 0.47)	(0.23, 0.54)	(0.55, 0.41)	(0.4, 0.47)
Information given by expert $u_{3}$	sub-attributes ${\dot{f}}_{i}$								
	${\dot{f}}_{1}$	${\dot{f}}_{2}$	${\dot{f}}_{3}$	${\dot{f}}_{4}$	${\dot{f}}_{5}$	${\dot{f}}_{6}$	${\dot{f}}_{7}$	${\dot{f}}_{8}$	${\dot{f}}_{9}$
Y^(1)^	(0.4, 0.33)	(0.44, 0.33)	(0.27, 0.4)	(0.26, 0.68)	(0.5, 0.41)	(0.28, 0.2)	(0.38, 0.59)	(0.21, 0.5)	(0.2, 0.4)
Y^(2)^	(0.79, 0.2)	(0.47, 0.33)	(0.43, 0.46)	(0.37, 0.49)	(0.21, 0.39)	(0.65, 0.21)	(0.24, 0.26)	(0.55, 0.2)	(0.36, 0.6)
Y^(3)^	(0.57, 0.27)	(0.2, 0.47)	(0.48, 0.24)	(0.55, 0.26)	(0.34, 0.46)	(0.37, 0.4)	(0.4, 0.43)	(0.31, 0.47)	(0.37, 0.34)
Y^(4)^	(0.64, 0.3)	(0.39, 0.52)	(0.49, 0.32)	(0.49, 0.28)	(0.43, 0.37)	(0.45, 0.29)	(0.35, 0.37)	(0.77, 0.17)	(0.51, 0.47)
Y^(5)^	(0.35, 0.51)	(0.55, 0.44)	(0.55, 0.38)	(0.46, 0.33)	(0.4, 0.51)	(0.54, 0.28)	(0.37, 0.29)	(0.23, 0.39)	(0.33, 0.55)

**Table 10:** The overall value of each alternative by the Cq-ROFHSEWA operator.

Alternatives	Cq-ROFHSEWA
Y^(1)^	(0.1321e^i2π0.0^, 0.754e^i2π0.0^)
Y^(2)^	(0.18e^i2π0.0^, 0.7458e^i2π0.0^)
Y^(3)^	(0.1685e^i2π0.0^, 0.7559e^i2π0.0^)
Y^(4)^	(0.1944e^i2π0.0^, 0.743e^i2π0.0^)
Y^(5)^	(0.1535e^i2π0.0^, 0.7815e^i2π0.0^)

**Table 11:** The overall value of each alternative by the Cq-ROFHSEWG operator.

Alternatives	Cq-ROFHSEWA
Y^(1)^	(0.7544e^i2π0.0^, 0.131e^i2π0.0^)
Y^(2)^	(0.8065e^i2π0.0^, 0.126e^i2π0.0^)
Y^(3)^	(0.7973e^i2π0.0^, 0.1322e^i2π0.0^)
Y^(4)^	(0.8242e^i2π0.0^, 0.1242e^i2π0.0^)
Y^(5)^	(0.7825e^i2π0.0^, 0.15e^i2π0.0^)

**Table 12:** Comparison between proposed method with existing methods for Example 5.

Methods	Similarity Values and Scores	Ranking Results
IFHSWA [30]	Ş^(1)^ = −0.5678, Ş^(2)^ = −0.4975, Ş^(3)^ = −0.5256, Ş^(4)^ = −0.4771, Ş^(5)^ = −0.5761	Y^(4)^ > Y^(2)^ > Y^(3)^ > Y^(1)^ > Y^(5)^
IFHSWG [30]	Ş^(1)^ = 0.5680, Ş^(2)^ = 0.6357, Ş^(3)^ = 0.6187, Ş^(4)^ = 0.6607, Ş^(5)^ = 0.5804	Y^(4)^ > Y^(2)^ > Y^(3)^ > Y^(5)^ > Y^(1)^
PFSWA [25]	Ş^(1)^ = 0.9973, Ş^(2)^ = 0.9988, Ş^(3)^ = 0.9983, Ş^(4)^ = 0.9993, Ş^(5)^ = 0.9979	Y^(4)^ > Y^(2)^ > Y^(3)^ > Y^(5)^ > Y^(1)^
PFSWG [25]	Ş^(1)^ = 0.9991, Ş^(2)^ = 0.9993, Ş^(3)^ = 0.9992, Ş^(4)^ = 0.9994, Ş^(5)^ = 0.9989	Y^(4)^ > Y^(2)^ > Y^(3)^ > Y^(1)^ > Y^(5)^
Cq-ROFWA, *q* = 1 [19]	Ş^(1)^ = 0.3581, Ş^(2)^ = 0.3756, Ş^(3)^ = 0.3686, Ş^(4)^ = 0.3807, Ş^(5)^ = 0.3560	Y^(4)^ > Y^(2)^ > Y^(3)^ > Y^(1)^ > Y^(5)^
Cq-ROFWG, *q* = 1 [19]	Ş^(1)^ = 0.6420, Ş^(2)^ = 0.6589, Ş^(3)^ = 0.6547, Ş^(4)^ = 0.6652, Ş^(5)^ = 0.6451	Y^(4)^ > Y^(2)^ > Y^(3)^ > Y^(5)^ > Y^(1)^
Proposed measure Cq-ROHSEWA for *q* = 1	Ş^(1)^ = 0.3445, Ş^(2)^ = 0.3585, Ş^(3)^ = 0.3531, Ş^(4)^ = 0.3629, Ş^(5)^ = 0.3430	Y^(4)^ > Y^(2)^ > Y^(3)^ > Y^(1)^ > Y^(5)^
Proposed measure Cq-ROHSEWG for *q* = 1	Ş^(1)^ = 0.6558, Ş^(2)^ = 0.6701, Ş^(3)^ = 0.6663, Ş^(4)^ = 0.6750, Ş^(5)^ = 0.6581	Y^(4)^ > Y^(2)^ > Y^(3)^ > Y^(5)^ > Y^(1)^

**Table 13:** Comparison between proposed method with existing methods for Example 4.

Methods	Similarity Values and Scores	Ranking Results
IFHSWA [30]	Ş^(1)^ = −0.5815, Ş^(2)^ = −0.5380, Ş^(3)^ = −0.5782, Ş^(4)^ = −0.4761, Ş^(5)^ = −0.4301	Y^(5)^ > Y^(4)^ > Y^(2)^ > Y^(3)^ > Y^(1)^
IFHSWG [30]	Ş^(1)^ = 0.4645, Ş^(2)^ = 0.5198, Ş^(3)^ = 0.5075, Ş^(4)^ = 0.5724, Ş^(5)^ = 0.6071	Y^(5)^ > Y^(4)^ > Y^(2)^ > Y^(3)^ > Y^(1)^
PFSWA [25]	Ş^(1)^ = 0.9901, Ş^(2)^ = 0.9909, Ş^(3)^ = 0.9947, Ş^(4)^ = 0.9943, Ş^(5)^ = 0.9954	Y^(5)^ > Y^(3)^ > Y^(4)^ > Y^(2)^ > Y^(1)^
PFSWG [25]	Ş^(1)^ = 0.9939, Ş^(2)^ = 0.9964, Ş^(3)^ = 0.9930, Ş^(4)^ = 0.9962, Ş^(5)^ = 0.9975	Y^(5)^ > Y^(2)^ > Y^(4)^ > Y^(1)^ > Y^(3)^
Cq-ROFWA, *q* = 3 [19]	Ş^(1)^ = 0.3839, Ş^(2)^ = 0.4063, Ş^(3)^ = 0.3566, Ş^(4)^ = 0.4155, Ş^(5)^ = 0.4292	Y^(5)^ > Y^(4)^ > Y^(2)^ > Y^(1)^ > Y^(3)^
Cq-ROFWG, *q* = 3 [19]	Ş^(1)^ = 0.5803, Ş^(2)^ = 0.5899, Ş^(3)^ = 0.6173, Ş^(4)^ = 0.6161, Ş^(5)^ = 0.6251	Y^(5)^ > Y^(3)^ > Y^(4)^ > Y^(2)^ > Y^(1)^
Proposed measure Cq-ROHSEWA for *q* = 3	Ş^(1)^ = 0.3616, Ş^(2)^ = 0.3815, Ş^(3)^ = 0.3399, Ş^(4)^ = 0.3897, Ş^(5)^ = 0.4032	Y^(5)^ > Y^(4)^ > Y^(2)^ > Y^(1)^ > Y^(3)^
Proposed measure Cq-ROHSEWG for *q* = 3	Ş^(1)^ = 0.6054, Ş^(2)^ = 0.6145, Ş^(3)^ = 0.6382, Ş^(4)^ = 0.6376, Ş^(5)^ = 0.6464	Y^(5)^ > Y^(3)^ > Y^(4)^ > Y^(2)^ > Y^(1)^

**Table 14:** Different ranking order from different methods.

Methods	Distributed Control System					Ranking Results
	Y^(1)^	Y^(2)^	Y^(3)^	Y^(4)^	Y^(5)^	
IFHSWA [30]	5	3	4	2	1	Y^(5)^ > Y^(4)^ > Y^(2)^ > Y^(3)^ > Y^(1)^
IFHSWG [30]	5	3	4	2	1	Y^(5)^ > Y^(4)^ > Y^(2)^ > Y^(3)^ > Y^(1)^
PFSWA [25]	5	4	2	3	1	Y^(5)^ > Y^(3)^ > Y^(4)^ > Y^(2)^ > Y^(1)^
PFSWG [25]	4	2	5	3	1	Y^(5)^ > Y^(2)^ > Y^(4)^ > Y^(1)^ > Y^(3)^
Cq-ROFWA, *q* = 3 [19]	4	3	5	2	1	Y^(5)^ > Y^(4)^ > Y^(2)^ > Y^(1)^ > Y^(3)^
Cq-ROFWG, *q* = 3 [19]	5	4	2	3	1	Y^(5)^ > Y^(3)^ > Y^(4)^ > Y^(2)^ > Y^(1)^
Proposed measure Cq-ROHSEWA for *q* = 3	4	3	5	2	1	Y^(5)^ > Y^(4)^ > Y^(2)^ > Y^(1)^ > Y^(3)^
Proposed measure Cq-ROHSEWG for *q* = 3	5	4	2	3	1	Y^(5)^ > Y^(3)^ > Y^(4)^ > Y^(2)^ > Y^(1)^

## Data Availability

Not applicable.

## Appendix A

**Proof** **of** **Theorem 2.** We prove this Theorem by mathematical introduction.

(1) When m=1,n=1, then clearly ${Ř}_{r}=1$, ${Ť}_{t}=1$ and the left side of Equation (11) becomes
  $$Cq-ROFHSEWA\left(A_{{}_{Q}11},A_{{}_{Q}12},\ldots ,A_{{}_{Q}rt}\right)=A_{{}_{Q}11}=\left(\varphi_{11}e^{i2\pi {Э}_{\varphi_{11}}},\psi_{11}e^{i2\pi {Э}_{\psi_{11}}}\right)$$

For the right side of Equation (11) we have

$$\begin{array}{l} =\left({\left(\frac{\left(1+\varphi_{11}^{q}\right)-\left(1-\varphi_{11}^{q}\right)}{\left(1+\varphi_{11}^{q}\right)+\left(1-\varphi_{11}^{q}\right)}\right)}^{\frac{1}{q}}e^{i2\pi {\left(\frac{\left(1+{Э}_{\varphi 11}^{q}\right)-\left(1-{Э}_{\varphi 11}^{q}\right)}{\left(1+{Э}_{\varphi 11}^{q}\right)+\left(1-{Э}_{\varphi 11}^{q}\right)}\right)}^{\frac{1}{q}}},\frac{2^{\frac{1}{q}}\left(\psi_{11}\right)}{{\left(\left(2-\psi_{11}^{q}\right)+\left(\psi_{11}^{q}\right)\right)}^{\frac{1}{q}}}e^{i2\pi \left(\frac{2^{\frac{1}{q}}\left({Э}_{\psi 11}\right)}{{\left(\left(2-{Э}_{\psi 11}^{q}\right)+\left({Э}_{\psi 11}^{q}\right)\right)}^{\frac{1}{q}}}\right)}\right)\\ =\left({\left(\frac{2\varphi_{11}^{q}}{2}\right)}^{\frac{1}{q}}e^{i2\pi {\left(\frac{2{Э}_{\varphi 11}^{q}}{2}\right)}^{\frac{1}{q}}},\frac{2^{\frac{1}{q}}\left(\psi_{11}\right)}{{\left(2\right)}^{\frac{1}{q}}}e^{i2\pi \left(\frac{2^{\frac{1}{q}}\left({Э}_{\psi 11}\right)}{{\left(2\right)}^{\frac{1}{q}}}\right)}\right)=\left(\varphi_{11}e^{i2\pi {Э}_{\varphi 11}},\psi_{11}e^{i2\pi {Э}_{\psi 11}}\right) \end{array}$$

(2) When m=1,n=2, then
  $${\overset{ˇ}{T}}_{1}\left({\overset{ˇ}{R}}_{1}A_{{}_{Q}11}\right)=\left(\begin{array}{cc} \; & {\left(\frac{{\left({\left(1+\varphi_{11}^{q}\right)}^{{\overset{ˇ}{R}}_{1}}\right)}^{{\overset{ˇ}{T}}_{1}}-{\left({\left(1-\varphi_{11}^{q}\right)}^{{\overset{ˇ}{R}}_{1}}\right)}^{{\overset{ˇ}{T}}_{1}}}{{\left({\left(1+\varphi_{11}^{q}\right)}^{{\overset{ˇ}{R}}_{1}}\right)}^{{\overset{ˇ}{T}}_{1}}+{\left({\left(1-\varphi_{11}^{q}\right)}^{{\overset{ˇ}{R}}_{1}}\right)}^{{\overset{ˇ}{T}}_{1}}}\right)}^{\frac{1}{q}}e^{i2\pi {\left(\frac{{\left({\left(1+{Э}_{\varphi 11}^{q}\right)}^{{\overset{ˇ}{R}}_{1}}\right)}^{{\overset{ˇ}{T}}_{1}}-{\left({\left(1-{Э}_{\varphi 11}^{q}\right)}^{{\overset{ˇ}{R}}_{1}}\right)}^{{\overset{ˇ}{T}}_{1}}}{{\left({\left(1+{Э}_{\varphi 11}^{q}\right)}^{{\overset{ˇ}{R}}_{1}}\right)}^{{\overset{ˇ}{T}}_{1}}+{\left({\left(1-{Э}_{\varphi 11}^{q}\right)}^{{\overset{ˇ}{R}}_{1}}\right)}^{{\overset{ˇ}{T}}_{1}}}\right)}^{\frac{1}{q}}},\\ \; & \frac{2^{\frac{1}{q}}{\left({\left(\psi_{11}\right)}^{{\overset{ˇ}{R}}_{1}}\right)}^{{\overset{ˇ}{T}}_{1}}}{{\left({\left({\left(2-\psi_{11}^{q}\right)}^{{\overset{ˇ}{R}}_{1}}\right)}^{{\overset{ˇ}{T}}_{1}}+{\left({\left(\psi_{11}^{q}\right)}^{{\overset{ˇ}{R}}_{1}}\right)}^{{\overset{ˇ}{T}}_{1}}\right)}^{\frac{1}{q}}}e^{i2\pi \left(\frac{2^{\frac{1}{q}}{\left({\left({Э}_{\psi 11}\right)}^{{\overset{ˇ}{R}}_{1}}\right)}^{{\overset{ˇ}{T}}_{1}}}{{\left({\left({\left(2-{Э}_{\psi 11}^{q}\right)}^{{\overset{ˇ}{R}}_{1}}\right)}^{{\overset{ˇ}{T}}_{1}}+{\left({\left({Э}_{\psi 11}^{q}\right)}^{{\overset{ˇ}{R}}_{1}}\right)}^{{\overset{ˇ}{T}}_{1}}\right)}^{\frac{1}{q}}}\right)} \end{array}\right),$$
  $${\overset{ˇ}{T}}_{2}\left({\overset{ˇ}{R}}_{1}A_{{}_{Q}12}\right)=\left(\begin{array}{cc} \; & {\left(\frac{{\left({\left(1+\varphi_{12}^{q}\right)}^{{\overset{ˇ}{R}}_{1}}\right)}^{{\overset{ˇ}{T}}_{2}}-{\left({\left(1-\varphi_{12}^{q}\right)}^{{\overset{ˇ}{R}}_{1}}\right)}^{{\overset{ˇ}{T}}_{2}}}{{\left({\left(1+\varphi_{12}^{q}\right)}^{{\overset{ˇ}{R}}_{1}}\right)}^{{\overset{ˇ}{T}}_{2}}+{\left({\left(1-\varphi_{12}^{q}\right)}^{{\overset{ˇ}{R}}_{1}}\right)}^{{\overset{ˇ}{T}}_{2}}}\right)}^{\frac{1}{q}}e^{i2\pi {\left(\frac{{\left({\left(1+{Э}_{\varphi 12}^{q}\right)}^{{\overset{ˇ}{R}}_{1}}\right)}^{{\overset{ˇ}{T}}_{2}}-{\left({\left(1-{Э}_{\varphi 12}^{q}\right)}^{{\overset{ˇ}{R}}_{1}}\right)}^{{\overset{ˇ}{T}}_{2}}}{{\left({\left(1+{Э}_{\varphi 12}^{q}\right)}^{{\overset{ˇ}{R}}_{1}}\right)}^{{\overset{ˇ}{T}}_{2}}+{\left({\left(1-{Э}_{\varphi 12}^{q}\right)}^{{\overset{ˇ}{R}}_{1}}\right)}^{{\overset{ˇ}{T}}_{2}}}\right)}^{\frac{1}{q}}},\\ \; & \frac{2^{\frac{1}{q}}{\left({\left(\psi_{12}\right)}^{{\overset{ˇ}{R}}_{1}}\right)}^{{\overset{ˇ}{T}}_{2}}}{{\left({\left({\left(2-\psi_{12}^{q}\right)}^{{\overset{ˇ}{R}}_{1}}\right)}^{{\overset{ˇ}{T}}_{2}}+{\left({\left(\psi_{12}^{q}\right)}^{{\overset{ˇ}{R}}_{1}}\right)}^{{\overset{ˇ}{T}}_{2}}\right)}^{\frac{1}{q}}}e^{i2\pi \left(\frac{2^{\frac{1}{q}}{\left({\left({Э}_{\psi 12}\right)}^{{\overset{ˇ}{R}}_{1}}\right)}^{{\overset{ˇ}{T}}_{2}}}{{\left({\left({\left(2-{Э}_{\psi 12}^{q}\right)}^{{\overset{ˇ}{R}}_{1}}\right)}^{{\overset{ˇ}{T}}_{2}}+{\left({\left({Э}_{\psi 12}^{q}\right)}^{{\overset{ˇ}{R}}_{1}}\right)}^{{\overset{ˇ}{T}}_{2}}\right)}^{\frac{1}{q}}}\right)} \end{array}\right),$$
  $$Cq-ROFHSEWA\left(A_{{}_{Q}11},A_{{}_{Q}12}\right)=\left({Ť}_{1}\left({Ř}_{1}A_{{}_{Q}11}\right)\right)⊕\left({Ť}_{2}\left({Ř}_{1}A_{{}_{Q}12}\right)\right)$$
  $$=\left(\begin{array}{cc} \; & {\left(\frac{{\prod_{t=1}^{2}\left({\left(1+\varphi_{1t}^{q}\right)}^{{\overset{ˇ}{R}}_{1}}\right)}^{{\overset{ˇ}{T}}_{t}}-{\prod_{t=1}^{2}\left({\left(1-\varphi_{1t}^{q}\right)}^{{\overset{ˇ}{R}}_{1}}\right)}^{{\overset{ˇ}{T}}_{t}}}{{\prod_{t=1}^{2}\left({\left(1+\varphi_{1t}^{q}\right)}^{{\overset{ˇ}{R}}_{1}}\right)}^{{\overset{ˇ}{T}}_{t}}+{\prod_{t=1}^{2}\left({\left(1-\varphi_{1t}^{q}\right)}^{{\overset{ˇ}{R}}_{1}}\right)}^{{\overset{ˇ}{T}}_{t}}}\right)}^{\frac{1}{q}}e^{i2\pi {\left(\frac{{\prod_{t=1}^{2}\left({\left(1+{Э}_{\varphi 1t}^{q}\right)}^{{\overset{ˇ}{R}}_{1}}\right)}^{{\overset{ˇ}{T}}_{t}}-{\prod_{t=1}^{2}\left({\left(1-{Э}_{\varphi 1t}^{q}\right)}^{{\overset{ˇ}{R}}_{1}}\right)}^{{\overset{ˇ}{T}}_{t}}}{{\prod_{t=1}^{2}\left({\left(1+{Э}_{\varphi 1t}^{q}\right)}^{{\overset{ˇ}{R}}_{1}}\right)}^{{\overset{ˇ}{T}}_{t}}+{\prod_{t=1}^{2}\left({\left(1-{Э}_{\varphi 1t}^{q}\right)}^{{\overset{ˇ}{R}}_{1}}\right)}^{{\overset{ˇ}{T}}_{t}}}\right)}^{\frac{1}{q}}},\\ \; & \frac{2^{\frac{1}{q}}{\prod_{t=1}^{2}\left({\left(\psi_{1t}\right)}^{{\overset{ˇ}{R}}_{1}}\right)}^{{\overset{ˇ}{T}}_{t}}}{{\left({\prod_{t=1}^{2}\left({\left(2-\psi_{1t}^{q}\right)}^{{\overset{ˇ}{R}}_{1}}\right)}^{{\overset{ˇ}{T}}_{t}}+{\prod_{t=1}^{2}\left({\left(\psi_{1t}^{q}\right)}^{{\overset{ˇ}{R}}_{1}}\right)}^{{\overset{ˇ}{T}}_{t}}\right)}^{\frac{1}{q}}}e^{i2\pi \left(\frac{2^{\frac{1}{q}}{\prod_{t=1}^{2}\left({\left({Э}_{\psi 1t}\right)}^{{\overset{ˇ}{R}}_{1}}\right)}^{{\overset{ˇ}{T}}_{t}}}{{\left({\prod_{t=1}^{2}\left({\left(2-{Э}_{\psi 1t}^{q}\right)}^{{\overset{ˇ}{R}}_{1}}\right)}^{{\overset{ˇ}{T}}_{t}}+{\prod_{t=1}^{2}\left({\left({Э}_{\psi 1t}^{q}\right)}^{{\overset{ˇ}{R}}_{1}}\right)}^{{\overset{ˇ}{T}}_{t}}\right)}^{\frac{1}{q}}}\right)} \end{array}\right)$$

Thus, Equation (11) is true for *m* = 1 and *n* = 2.

(3) Suppose that Equation (11) holds for m=k,n=ℓ. Then, we have
  $$\begin{array}{l} Cq-ROFHSEWA\left(A_{{}_{Q}11},A_{{}_{Q}12},\ldots ,A_{{}_{Q}kl}\right)=\\ \left({Ť}_{1}\left({Ř}_{1}A_{{}_{Q}11}\right)\right)⊕\left({Ť}_{2}\left({Ř}_{1}A_{{}_{Q}12}\right)\right)⊕\ldots ⊕\left({Ť}_{l}\left({Ř}_{k}A_{{}_{Q}kl}\right)\right)={⊕}_{t=1}^{l}{Ť}_{t}\left({⊕}_{r=1}^{k}{Ř}_{r}A_{{}_{Q}rt}\right) \end{array}$$
  $$=\left(\begin{array}{cc} \; & {\left(\frac{{\prod_{t=1}^{l}\left(\prod_{r=1}^{k}{\left(1+\varphi_{rt}^{q}\right)}^{{\overset{ˇ}{R}}_{r}}\right)}^{{\overset{ˇ}{T}}_{t}}-{\prod_{t=1}^{l}\left(\prod_{r=1}^{k}{\left(1-\varphi_{rt}^{q}\right)}^{{\overset{ˇ}{R}}_{r}}\right)}^{{\overset{ˇ}{T}}_{t}}}{{\prod_{t=1}^{l}\left(\prod_{r=1}^{k}{\left(1+\varphi_{rt}^{q}\right)}^{{\overset{ˇ}{R}}_{r}}\right)}^{{\overset{ˇ}{T}}_{t}}+{\prod_{t=1}^{l}\left(\prod_{r=1}^{k}{\left(1-\varphi_{rt}^{q}\right)}^{{\overset{ˇ}{R}}_{r}}\right)}^{{\overset{ˇ}{T}}_{t}}}\right)}^{\frac{1}{q}}e^{i2\pi {\left(\frac{{\prod_{t=1}^{\ell }\left(\prod_{r=1}^{k}{\left(1+{Э}_{\varphi_{rt}}^{q}\right)}^{{\overset{ˇ}{R}}_{r}}\right)}^{{\overset{ˇ}{T}}_{t}}-{\prod_{t=1}^{\ell }\left(\prod_{r=1}^{k}{\left(1-{Э}_{\varphi_{rt}}^{q}\right)}^{{\overset{ˇ}{R}}_{r}}\right)}^{{\overset{ˇ}{T}}_{t}}}{{\prod_{t=1}^{\ell }\left(\prod_{r=1}^{k}{\left(1+{Э}_{\varphi_{rt}}^{q}\right)}^{{\overset{ˇ}{R}}_{r}}\right)}^{{\overset{ˇ}{T}}_{t}}+{\prod_{t=1}^{\ell }\left(\prod_{r=1}^{k}{\left(1-{Э}_{\varphi_{rt}}^{q}\right)}^{{\overset{ˇ}{R}}_{r}}\right)}^{{\overset{ˇ}{T}}_{t}}}\right)}^{\frac{1}{q}}},\\ \; & \frac{2^{\frac{1}{q}}{\prod_{t=1}^{l}\left(\prod_{r=1}^{k}{\left(\psi_{rt}\right)}^{{\overset{ˇ}{R}}_{r}}\right)}^{{\overset{ˇ}{T}}_{t}}}{{\left({\prod_{t=1}^{l}\left(\prod_{r=1}^{k}{\left(2-\psi_{rt}^{q}\right)}^{{\overset{ˇ}{R}}_{r}}\right)}^{{\overset{ˇ}{T}}_{t}}+{\prod_{t=1}^{l}\left(\prod_{r=1}^{k}{\left(\psi_{rt}^{q}\right)}^{{\overset{ˇ}{R}}_{r}}\right)}^{{\overset{ˇ}{T}}_{t}}\right)}^{\frac{1}{q}}}e^{i2\pi \left(\frac{2^{\frac{1}{q}}{\prod_{ΐ=1}^{\ell }\left(\prod_{r=1}^{k}{\left({Э}_{\psi_{rt}}\right)}^{{\overset{ˇ}{R}}_{r}}\right)}^{{\overset{ˇ}{T}}_{t}}}{{\left({\prod_{t=1}^{l}\left(\prod_{r=1}^{k}{\left(2{-}_{\psi_{rt}}^{q}\right)}^{{\overset{ˇ}{R}}_{r}}\right)}^{{\overset{ˇ}{T}}_{t}}+{\prod_{t=1}^{l}\left(\prod_{r=1}^{k}{\left({Э}_{\psi_{rt}}^{q}\right)}^{{\overset{ˇ}{R}}_{r}}\right)}^{{\overset{ˇ}{T}}_{t}}\right)}^{\frac{1}{q}}}\right)} \end{array}\right)$$
  $$Cq-ROFHSEWA\left(A_{{}_{Q}11},A_{{}_{Q}12},\ldots ,A_{{}_{Q}\left(k+1\right)\left(l+1\right)}\right)=Cq-ROFHSEWA\left(A_{{}_{Q}11},A_{{}_{Q}12},\ldots ,A_{{}_{Q}kl}\right)⊕A_{{}_{Q}\left(k+1\right)\left(l+1\right)}$$
  $$=\left(\begin{array}{cc} \; & {\left(\frac{{\prod_{t=1}^{l}\left(\prod_{r=1}^{k}{\left(1+\varphi_{rt}^{q}\right)}^{{\overset{ˇ}{R}}_{r}}\right)}^{{\overset{ˇ}{T}}_{t}}-{\prod_{t=1}^{l}\left(\prod_{r=1}^{k}{\left(1-\varphi_{rt}^{q}\right)}^{{\overset{ˇ}{R}}_{r}}\right)}^{{\overset{ˇ}{T}}_{t}}}{{\prod_{t=1}^{l}\left(\prod_{r=1}^{k}{\left(1+\varphi_{rt}^{q}\right)}^{{\overset{ˇ}{R}}_{r}}\right)}^{{\overset{ˇ}{T}}_{t}}+{\prod_{t=1}^{l}\left(\prod_{r=1}^{k}{\left(1-\varphi_{rt}^{q}\right)}^{{\overset{ˇ}{R}}_{r}}\right)}^{{\overset{ˇ}{T}}_{t}}}\right)}^{\frac{1}{q}}e^{i2\pi {\left(\frac{{\prod_{t=1}^{l}\left(\prod_{r=1}^{k}{\left(1+{Э}_{\varphi_{rt}}^{q}\right)}^{{\overset{ˇ}{R}}_{r}}\right)}^{{\overset{ˇ}{T}}_{t}}-{\prod_{t=1}^{l}\left(\prod_{r=1}^{k}{\left(1-{Э}_{\varphi_{rt}}^{q}\right)}^{{\overset{ˇ}{R}}_{r}}\right)}^{{\overset{ˇ}{T}}_{t}}}{{\prod_{t=1}^{l}\left(\prod_{r=1}^{k}{\left(1+{Э}_{\varphi_{rt}}^{q}\right)}^{{\overset{ˇ}{R}}_{r}}\right)}^{{\overset{ˇ}{T}}_{t}}+{\prod_{t=1}^{l}\left(\prod_{r=1}^{k}{\left(1-{Э}_{\varphi_{rt}}^{q}\right)}^{{\overset{ˇ}{R}}_{r}}\right)}^{{\overset{ˇ}{T}}_{t}}}\right)}^{\frac{1}{q}}},\\ \; & \frac{2^{\frac{1}{q}}{\prod_{t=1}^{l}\left(\prod_{r=1}^{k}{\left(\psi_{rt}\right)}^{{\overset{ˇ}{R}}_{r}}\right)}^{{\overset{ˇ}{T}}_{t}}}{{\left({\prod_{t=1}^{l}\left(\prod_{r=1}^{k}{\left(2-\psi_{rt}^{q}\right)}^{{\overset{ˇ}{R}}_{r}}\right)}^{{\overset{ˇ}{T}}_{t}}+{\prod_{t=1}^{l}\left(\prod_{r=1}^{k}{\left(\psi_{rt}^{q}\right)}^{{\overset{ˇ}{R}}_{r}}\right)}^{{\overset{ˇ}{T}}_{t}}\right)}^{\frac{1}{q}}}e^{i2\pi \left(\frac{2^{\frac{1}{q}}{\prod_{t=1}^{\ell }\left(\prod_{r=1}^{k}{\left({Э}_{\psi_{rt}}\right)}^{{\overset{ˇ}{R}}_{r}}\right)}^{{\overset{ˇ}{T}}_{t}}}{{\left({\prod_{t=1}^{l}\left(\prod_{r=1}^{k}{\left(2-{Э}_{\psi_{rt}}^{q}\right)}^{{\overset{ˇ}{R}}_{r}}\right)}^{{\overset{ˇ}{T}}_{t}}+{\prod_{t=1}^{l}\left(\prod_{r=1}^{k}{\left({Э}_{\psi_{rt}}^{q}\right)}^{{\overset{ˇ}{R}}_{r}}\right)}^{{\overset{ˇ}{T}}_{t}}\right)}^{\frac{1}{q}}}\right)} \end{array}\right)$$
  $$⊕\left(\begin{array}{cc} \; & {\left(\frac{{\left({\left(1+\varphi_{\left(k+1\right)\left(l+1\right)}^{q}\right)}^{{\overset{ˇ}{R}}_{k+1}}\right)}^{{\overset{ˇ}{T}}_{l+1}}-{\left({\left(1-\varphi_{\left(k+1\right)\left(l+1\right)}^{q}\right)}^{{\overset{ˇ}{R}}_{k+1}}\right)}^{{\overset{ˇ}{T}}_{l+1}}}{{\left({\left(1+\varphi_{\left(k+1\right)\left(l+1\right)}^{q}\right)}^{{\overset{ˇ}{R}}_{k+1}}\right)}^{{\overset{ˇ}{T}}_{l+1}}+{\left({\left(1-\varphi_{\left(k+1\right)\left(l+1\right)}^{q}\right)}^{{\overset{ˇ}{R}}_{k+1}}\right)}^{{\overset{ˇ}{T}}_{l+1}}}\right)}^{\frac{1}{q}}e^{i2\pi {\left(\frac{{\left({\left(1+{Э}_{\varphi \left(k+1\right)\left(l+1\right)}^{q}\right)}^{{\overset{ˇ}{R}}_{k+1}}\right)}^{{\overset{ˇ}{T}}_{l+1}}-{\left({\left(1-{Э}_{\varphi \left(k+1\right)\left(l+1\right)}^{q}\right)}^{{\overset{ˇ}{R}}_{k+1}}\right)}^{{\overset{ˇ}{T}}_{l+1}}}{{\left({\left(1+{Э}_{\varphi \left(k+1\right)\left(l+1\right)}^{q}\right)}^{{\overset{ˇ}{R}}_{k+1}}\right)}^{{\overset{ˇ}{T}}_{l+1}}+{\left({\left(1-{Э}_{\varphi \left(k+1\right)\left(l+1\right)}^{q}\right)}^{{\overset{ˇ}{R}}_{k+1}}\right)}^{{\overset{ˇ}{T}}_{l+1}}}\right)}^{\frac{1}{q}}},\\ \; & \frac{2^{\frac{1}{q}}{\left({\left(\psi_{\left(k+1\right)\left(l+1\right)}\right)}^{{\overset{ˇ}{R}}_{k+1}}\right)}^{{\overset{ˇ}{T}}_{l+1}}}{{\left({\left({\left(2-\psi_{\left(k+1\right)\left(l+1\right)}^{q}\right)}^{{\overset{ˇ}{R}}_{k+1}}\right)}^{{\overset{ˇ}{T}}_{l+1}}+{\left({\left(\psi_{\left(k+1\right)\left(l+1\right)}^{q}\right)}^{{\overset{ˇ}{R}}_{k+1}}\right)}^{{\overset{ˇ}{T}}_{l+1}}\right)}^{\frac{1}{q}}}e^{i2\pi \left(\frac{2^{\frac{1}{q}}{\left({\left({Э}_{\psi \left(k+1\right)\left(l+1\right)}\right)}^{{\overset{ˇ}{R}}_{k+1}}\right)}^{{\overset{ˇ}{T}}_{l+1}}}{{\left({\left({\left(2-{Э}_{\psi \left(k+1\right)\left(l+1\right)}^{q}\right)}^{{\overset{ˇ}{R}}_{k+1}}\right)}^{{\overset{ˇ}{T}}_{l+1}}+{\left({\left({Э}_{\psi \left(k+1\right)\left(l+1\right)}^{q}\right)}^{{\overset{ˇ}{R}}_{k+1}}\right)}^{{\overset{ˇ}{T}}_{l+1}}\right)}^{\frac{1}{q}}}\right)} \end{array}\right)$$
  $$=\left(\begin{array}{cc} \; & {\left(\frac{{\prod_{t=1}^{l+1}\left(\prod_{r=1}^{k+1}{\left(1+\varphi_{rt}^{q}\right)}^{{\overset{ˇ}{R}}_{r}}\right)}^{{\overset{ˇ}{T}}_{t}}-{\prod_{t=1}^{l+1}\left(\prod_{r=1}^{k+1}{\left(1-\varphi_{rt}^{q}\right)}^{{\overset{ˇ}{R}}_{r}}\right)}^{{\overset{ˇ}{T}}_{t}}}{{\prod_{t=1}^{l+1}\left(\prod_{r=1}^{k+1}{\left(1+\varphi_{rt}^{q}\right)}^{{\overset{ˇ}{R}}_{r}}\right)}^{{\overset{ˇ}{T}}_{t}}+{\prod_{t=1}^{l+1}\left(\prod_{r=1}^{k+1}{\left(1-\varphi_{rt}^{q}\right)}^{{\overset{ˇ}{R}}_{r}}\right)}^{{\overset{ˇ}{T}}_{t}}}\right)}^{\frac{1}{q}}e^{i2\pi {\left(\frac{{\prod_{t=1}^{l+1}\left(\prod_{r=1}^{k+1}{\left(1+{Э}_{\varphi_{rt}}^{q}\right)}^{{\overset{ˇ}{R}}_{r}}\right)}^{{\overset{ˇ}{T}}_{t}}-{\prod_{t=1}^{l+1}\left(\prod_{r=1}^{k+1}{\left(1-{Э}_{\varphi_{rt}}^{q}\right)}^{{\overset{ˇ}{R}}_{r}}\right)}^{{\overset{ˇ}{T}}_{t}}}{{\prod_{t=1}^{l+1}\left(\prod_{r=1}^{k+1}{\left(1+{Э}_{\varphi_{rt}}^{q}\right)}^{{\overset{ˇ}{R}}_{r}}\right)}^{{\overset{ˇ}{T}}_{t}}+{\prod_{t=1}^{l+1}\left(\prod_{r=1}^{k+1}{\left(1-{Э}_{\varphi_{rt}}^{q}\right)}^{{\overset{ˇ}{R}}_{r}}\right)}^{{\overset{ˇ}{T}}_{t}}}\right)}^{\frac{1}{q}}},\\ \; & \frac{2^{\frac{1}{q}}{\prod_{t=1}^{l+1}\left(\prod_{r=1}^{k+1}{\left(\psi_{rt}\right)}^{{\overset{ˇ}{R}}_{r}}\right)}^{{\overset{ˇ}{T}}_{t}}}{{\left({\prod_{t=1}^{l+1}\left(\prod_{r=1}^{k+1}{\left(2-\psi_{rt}^{q}\right)}^{{\overset{ˇ}{R}}_{r}}\right)}^{{\overset{ˇ}{T}}_{t}}+{\prod_{t=1}^{l+1}\left(\prod_{r=1}^{k+1}{\left(\psi_{rt}^{q}\right)}^{{\overset{ˇ}{R}}_{r}}\right)}^{{\overset{ˇ}{T}}_{t}}\right)}^{\frac{1}{q}}}e^{i2\pi \left(\frac{2^{\frac{1}{q}}{\prod_{t=1}^{l+1}\left(\prod_{r=1}^{k+1}{\left({Э}_{\psi_{rt}}\right)}^{{\overset{ˇ}{R}}_{r}}\right)}^{{\overset{ˇ}{T}}_{t}}}{{\left({\prod_{t=1}^{l+1}\left(\prod_{r=1}^{k+1}{\left(2-{Э}_{\psi_{rt}}^{q}\right)}^{{\overset{ˇ}{R}}_{r}}\right)}^{{\overset{ˇ}{T}}_{t}}+{\prod_{t=1}^{l+1}\left(\prod_{r=1}^{k+1}{\left({Э}_{\psi_{rt}}^{q}\right)}^{{\overset{ˇ}{R}}_{r}}\right)}^{{\overset{ˇ}{T}}_{t}}\right)}^{\frac{1}{q}}}\right)} \end{array}\right)$$

Hence, the result holds for *m* = *k* + 1, *n* = *ℓ* + 1 and, therefore, holds true for all values of *m*, *n*.

**Proof** **of** **Theorem 3.** (1) For $A_{{}_{Q}rt}=A_{{}_{Q}}=\left(\varphi_{Q}e^{i2\pi {Э}_{\varphi }},\psi_{Q}e^{i2\pi {Э}_{\psi }}\right)$ (r = 1, 2, …, m, t = 1, 2, …, n), by Definition (7), the result is yielded below: $Cq-ROFHSEWA\left(A_{{}_{Q}11},A_{{}_{Q}12},\ldots ,A_{{}_{Q}rt}\right)={⊕}_{t=1}^{n}{Ť}_{t}\left({⊕}_{r=1}^{m}{Ř}_{r}A_{{}_{Q}rt}\right)$

$$=\left(\begin{array}{cc} \; & {\left(\frac{{\prod_{t=1}^{n}\left(\prod_{r=1}^{m}{\left(1+\varphi_{rt}^{q}\right)}^{{\overset{ˇ}{R}}_{r}}\right)}^{{\overset{ˇ}{T}}_{t}}-{\prod_{t=1}^{n}\left(\prod_{r=1}^{m}{\left(1-\varphi_{rt}^{q}\right)}^{{\overset{ˇ}{R}}_{r}}\right)}^{{\overset{ˇ}{T}}_{t}}}{{\prod_{t=1}^{n}\left(\prod_{r=1}^{m}{\left(1+\varphi_{rt}^{q}\right)}^{{\overset{ˇ}{R}}_{r}}\right)}^{{\overset{ˇ}{T}}_{t}}+{\prod_{t=1}^{n}\left(\prod_{r=1}^{m}{\left(1-\varphi_{rt}^{q}\right)}^{{\overset{ˇ}{R}}_{r}}\right)}^{{\overset{ˇ}{T}}_{t}}}\right)}^{\frac{1}{q}}e^{i2\pi {\left(\frac{{\prod_{t=1}^{n}\left(\prod_{r=1}^{m}{\left(1+{Э}_{\varphi_{rt}}^{q}\right)}^{{\overset{ˇ}{R}}_{r}}\right)}^{{\overset{ˇ}{T}}_{t}}-{\prod_{t=1}^{n}\left(\prod_{r=1}^{m}{\left(1-{Э}_{\varphi_{rt}}^{q}\right)}^{{\overset{ˇ}{R}}_{r}}\right)}^{{\overset{ˇ}{T}}_{t}}}{{\prod_{t=1}^{n}\left(\prod_{r=1}^{m}{\left(1+{Э}_{\varphi_{rt}}^{q}\right)}^{{\overset{ˇ}{R}}_{r}}\right)}^{{\overset{ˇ}{T}}_{t}}+{\prod_{t=1}^{n}\left(\prod_{r=1}^{m}{\left(1-{Э}_{\varphi_{rt}}^{q}\right)}^{{\overset{ˇ}{R}}_{r}}\right)}^{{\overset{ˇ}{T}}_{t}}}\right)}^{\frac{1}{q}}},\\ \; & \frac{2^{\frac{1}{q}}{\prod_{t=1}^{n}\left(\prod_{r=1}^{m}{\left(\psi_{rt}\right)}^{{\overset{ˇ}{R}}_{r}}\right)}^{{\overset{ˇ}{T}}_{t}}}{{\left({\prod_{t=1}^{n}\left(\prod_{r=1}^{m}{\left(2-\psi_{rt}^{q}\right)}^{{\overset{ˇ}{R}}_{r}}\right)}^{{\overset{ˇ}{T}}_{t}}+{\prod_{t=1}^{n}\left(\prod_{r=1}^{m}{\left(\psi_{rt}^{q}\right)}^{{\overset{ˇ}{R}}_{r}}\right)}^{{\overset{ˇ}{T}}_{t}}\right)}^{\frac{1}{q}}}e^{i2\pi \left(\frac{2^{\frac{1}{q}}{\prod_{t=1}^{n}\left(\prod_{r=1}^{m}{\left({Э}_{\psi_{rt}}\right)}^{{\overset{ˇ}{R}}_{r}}\right)}^{{\overset{ˇ}{T}}_{t}}}{{\left({\prod_{t=1}^{n}\left(\prod_{r=1}^{m}{\left(2-{Э}_{\psi_{rt}}^{q}\right)}^{{\overset{ˇ}{R}}_{r}}\right)}^{{\overset{ˇ}{T}}_{t}}+{\prod_{t=1}^{n}\left(\prod_{r=1}^{m}{\left({Э}_{\psi_{rt}}^{q}\right)}^{{\overset{ˇ}{R}}_{r}}\right)}^{{\overset{ˇ}{T}}_{t}}\right)}^{\frac{1}{q}}}\right)} \end{array}\right)$$

$$=\left(\begin{array}{cc} \; & {\left(\frac{{\left({\left(1+\varphi^{q}\right)}^{\sum_{r=1}^{m}{\overset{ˇ}{R}}_{r}}\right)}^{\sum_{t=1}^{n}{\overset{ˇ}{T}}_{t}}-{\left({\left(1-\varphi^{q}\right)}^{\sum_{r=1}^{m}{\overset{ˇ}{R}}_{r}}\right)}^{\sum_{t=1}^{n}{\overset{ˇ}{T}}_{t}}}{{\left({\left(1+\varphi^{q}\right)}^{\sum_{r=1}^{m}{\overset{ˇ}{R}}_{r}}\right)}^{\sum_{t=1}^{n}{\overset{ˇ}{T}}_{t}}+{\left({\left(1-\varphi^{q}\right)}^{\sum_{r=1}^{m}{\overset{ˇ}{R}}_{r}}\right)}^{\sum_{t=1}^{n}{\overset{ˇ}{T}}_{t}}}\right)}^{\frac{1}{q}}e^{i2\pi {\left(\frac{{\left({\left(1+{Э}_{\varphi }^{q}\right)}^{\sum_{r=1}^{m}{\overset{ˇ}{R}}_{r}}\right)}^{\sum_{t=1}^{n}{\overset{ˇ}{T}}_{t}}-{\left({\left(1-{Э}_{\varphi }^{q}\right)}^{\sum_{r=1}^{m}{\overset{ˇ}{R}}_{r}}\right)}^{\sum_{t=1}^{n}{\overset{ˇ}{T}}_{t}}}{{\left({\left(1+{Э}_{\varphi }^{q}\right)}^{\sum_{r=1}^{m}{\overset{ˇ}{R}}_{r}}\right)}^{\sum_{t=1}^{n}{\overset{ˇ}{T}}_{t}}+{\left({\left(1-{Э}_{\varphi }^{q}\right)}^{\sum_{r=1}^{m}{\overset{ˇ}{R}}_{r}}\right)}^{\sum_{t=1}^{n}{\overset{ˇ}{T}}_{t}}}\right)}^{\frac{1}{q}}},\\ \; & \frac{2^{\frac{1}{q}}{\left({\left(\psi \right)}^{\sum_{r=1}^{m}{\overset{ˇ}{R}}_{r}}\right)}^{\sum_{t=1}^{n}{\overset{ˇ}{T}}_{t}}}{{\left({\left({\left(2-\psi^{q}\right)}^{\sum_{r=1}^{m}{\overset{ˇ}{R}}_{r}}\right)}^{\sum_{t=1}^{n}{\overset{ˇ}{T}}_{t}}+{\left({\left(\psi^{q}\right)}^{\sum_{r=1}^{m}{\overset{ˇ}{R}}_{r}}\right)}^{\sum_{t=1}^{n}{\overset{ˇ}{T}}_{t}}\right)}^{\frac{1}{q}}}e^{i2\pi \left(\frac{2^{\frac{1}{q}}{\left({\left({Э}_{\psi }\right)}^{\sum_{r=1}^{m}{\overset{ˇ}{R}}_{r}}\right)}^{\sum_{t=1}^{n}{\overset{ˇ}{T}}_{t}}}{{\left({\left({\left(2-{Э}_{\psi }^{q}\right)}^{\sum_{r=1}^{m}{\overset{ˇ}{R}}_{r}}\right)}^{\sum_{t=1}^{n}{\overset{ˇ}{T}}_{t}}+{\left({\left({Э}_{\psi }^{q}\right)}^{\sum_{r=1}^{m}{\overset{ˇ}{R}}_{r}}\right)}^{\sum_{t=1}^{n}{\overset{ˇ}{T}}_{t}}\right)}^{\frac{1}{q}}}\right)} \end{array}\right)$$

$$=\left(\begin{array}{cc} \; & {\left(\frac{\left(1+\varphi^{q}\right)-\left(1-\varphi^{q}\right)}{\left(1+\varphi^{q}\right)+\left(1-\varphi^{q}\right)}\right)}^{\frac{1}{q}}e^{i2\pi {\left(\frac{\left(1+{Э}_{\varphi }^{q}\right)-\left(1-{Э}_{\varphi }^{q}\right)}{\left(1+{Э}_{\varphi }^{q}\right)+\left(1-{Э}_{\varphi }^{q}\right)}\right)}^{\frac{1}{q}}},\\ \; & \frac{2^{\frac{1}{q}}\psi }{{\left(\left(2-\psi^{q}\right)+\psi^{q}\right)}^{\frac{1}{q}}}e^{i2\pi \left(\frac{2^{\frac{1}{q}}{Э}_{\psi }}{{\left(\left(2-{Э}_{\psi }^{q}\right)+{Э}_{\psi }^{q}\right)}^{\frac{1}{q}}}\right)} \end{array}\right)=\left(\begin{array}{cc} \; & {\left(\frac{2\varphi^{q}}{2}\right)}^{\frac{1}{q}}e^{i2\pi {\left(\frac{2{Э}_{\varphi }^{q}}{2}\right)}^{\frac{1}{q}}},\\ \; & \frac{2^{\frac{1}{q}}\psi }{{\left(2\right)}^{\frac{1}{q}}}e^{i2\pi \left(\frac{2^{\frac{1}{q}}{Э}_{\psi }}{{\left(2\right)}^{\frac{1}{q}}}\right)} \end{array}\right)=\left(\varphi_{Q}e^{i2\pi {Э}_{\varphi }},\psi_{Q}e^{i2\pi {Э}_{\psi }}\right)=A_{{}_{Q}}$$

(2) There exists the inequality $A_{{}_{Q}}^{-}\leq A_{{}_{Q}rt}\leq A_{{}_{Q}}^{+}$ when $A_{{}_{Q}}^{-}=\left(\min\varphi_{rt}e^{i2\pi \min{Э}_{\varphi_{rt}}},\max\psi_{rt}e^{i2\pi \max{Э}_{\psi_{rt}}}\right)$ and $A_{{}_{Q}}^{+}=\left(\max\varphi_{rt}e^{i2\pi \max{Э}_{\varphi_{rt}}},\min\psi_{rt}e^{i2\pi \min{Э}_{\psi_{rt}}}\right)$. Thus, there also exists ${⊕}_{t=1}^{l}{Ť}_{t}\left({⊕}_{r=1}^{k}{Ř}_{r}A_{{}_{Q}}^{-}\right)\subseteq {⊕}_{t=1}^{l}{Ť}_{t}\left({⊕}_{r=1}^{k}{Ř}_{r}A_{{}_{Q}rt}\right)\subseteq {⊕}_{t=1}^{l}{Ť}_{t}\left({⊕}_{r=1}^{k}{Ř}_{r}A_{{}_{Q}}^{+}\right)$. Then, the inequality $A_{{}_{Q}}^{-}\subseteq {⊕}_{t=1}^{l}{Ť}_{t}\left({⊕}_{r=1}^{k}{Ř}_{r}A_{{}_{Q}rt}\right)\subseteq A_{{}_{Q}}^{+}$ can be kept regarding the above property (1); i.e., there is $A_{{}_{Q}}^{-}\subseteq Cq-ROFHSEWA\left(A_{{}_{Q}11},A_{{}_{Q}12},\ldots ,A_{{}_{Q}rt}\right)\subseteq A_{{}_{Q}}^{+}$. (3) For $A_{{}_{Q}rt}\subseteq {\ddot{A}}_{{}_{Q}rt}$, there is the inequality ${⊕}_{t=1}^{l}{Ť}_{t}\left({⊕}_{r=1}^{k}{Ř}_{r}A_{{}_{Q}rt}\right)\subseteq {⊕}_{t=1}^{l}{Ť}_{t}\left({⊕}_{r=1}^{k}{Ř}_{r}{\ddot{A}}_{{}_{Q}rt}\right)$; i.e., $Cq-ROFHSEWA\left(A_{{}_{Q}11},A_{{}_{Q}12},\ldots ,A_{{}_{Q}rt}\right)\subseteq Cq-ROFHSEWA\left({\ddot{A}}_{{}_{Q}11},{\ddot{A}}_{{}_{Q}12},\ldots ,{\ddot{A}}_{{}_{Q}rt}\right)$ exists. Therefore, all the above properties are true.
